# Investigating differences in village-level heterogeneity of malaria infection and household risk factors in Papua New Guinea

**DOI:** 10.1038/s41598-021-95959-8

**Published:** 2021-08-16

**Authors:** Desmond Gul, Daniela Rodríguez-Rodríguez, Elma Nate, Alma Auwan, Mary Salib, Lina Lorry, John B. Keven, Michelle Katusele, Jason Rosado, Natalie Hofmann, Maria Ome-Kaius, Cristian Koepfli, Ingrid Felger, James W. Kazura, Manuel W. Hetzel, Ivo Mueller, Stephan Karl, Archie C. A. Clements, Freya J. I. Fowkes, Moses Laman, Leanne J. Robinson

**Affiliations:** 1 Burnet Institute, Melbourne, Australia; 2 School of Public Health and Preventive Medicine, Monash University, Melbourne, Australia; 3 Swiss Tropical and Public Health Institute, Basel, Switzerland; 4 University of Basel, Basel, Switzerland; 5 Papua New Guinea Institute of Medical Research, Madang, Papua New Guinea; 6 Department of Microbiology and Molecular Genetics, Michigan State University, Michigan, USA; 7 Unit of Malaria: Parasites and Hosts, Institut Pasteur, Paris, France; 8 Sorbonne University, Paris, France; 9 University of Notre Dame, Notre Dame, IN USA; 10 Walter and Eliza Hall Institute of Medical Research, Melbourne, Australia; 11 Case Western Reserve University, Cleveland, OH USA; 12 James Cook University, Cairns, Australia; 13 Curtin University, Perth, Australia; 14 Telethon Kids Institute, Perth, Australia; 15 Melbourne University, Melbourne, Australia

**Keywords:** Malaria, Epidemiology, Risk factors

## Abstract

Malaria risk is highly heterogeneous. Understanding village and household-level spatial heterogeneity of malaria risk can support a transition to spatially targeted interventions for malaria elimination. This analysis uses data from cross-sectional prevalence surveys conducted in 2014 and 2016 in two villages (Megiar and Mirap) in Papua New Guinea. Generalised additive modelling was used to characterise spatial heterogeneity of malaria risk and investigate the contribution of individual, household and environmental-level risk factors. Following a period of declining malaria prevalence, the prevalence of *P. falciparum* increased from 11.4 to 19.1% in Megiar and 12.3 to 28.3% in Mirap between 2014 and 2016, with focal hotspots observed in these villages in 2014 and expanding in 2016. Prevalence of *P. vivax* was similar in both years (20.6% and 18.3% in Megiar, 22.1% and 23.4% in Mirap) and spatial risk heterogeneity was less apparent compared to *P. falciparum*. Within-village hotspots varied by *Plasmodium* species across time and between villages. In Megiar, the adjusted odds ratio (AOR) of infection could be partially explained by household factors that increase risk of vector exposure, such as collecting outdoor surface water as a main source of water. In Mirap, increased AOR overlapped with proximity to densely vegetated areas of the village. The identification of household and environmental factors associated with increased spatial risk may serve as useful indicators of transmission hotspots and inform the development of tailored approaches for malaria control.

## Introduction

Increased investment towards the scale-up of malaria control interventions led to a global decline in malaria incidence between 2010 and 2018, from 72 to 57 cases per 1000 population at risk^1^. The main interventions are vector control with long-lasting insecticide treated bed nets (LLINs), indoor residual spraying (IRS), rapid diagnostic tests (RDTs) for prompt and improved case diagnosis, and treatment with artemisinin combination therapy (ACT)^2^. Although uniform application of these standard control methods has been effective in reducing the burden of malaria, global progress has stalled and some countries are reporting an increase in malaria incidence^1^.

As endemic countries make progress towards the goal of malaria elimination, malaria transmission has been observed to become more heterogenous^3,4^. The individual risk of a *Plasmodium *spp. infection and heterogeneity in that risk is influenced by climate, physical geography, land use, human behaviour and social factors at village or household level, and variations in health service delivery. As malaria transmission decreases, infections tend to become clustered in localised communities, households, or groups of people. In a geospatial sense, these clusters, known as “hotspots”, are defined as areas where there is a higher than expected risk of malaria *Plasmodium* spp. infections compared to surrounding areas, reflecting a higher than average level of malaria transmission intensity. Hotspots have been reported in several studies at various spatial scales from province or district levels right down to village and household levels^4–9^.

WHO recommends stratification at spatial scales that are commensurate with transmission levels so that appropriate targeted interventions can be achieved^2^. However, the spatial scale chosen should also be determined by local epidemiology, data availability and programmatic objectives. As countries move towards elimination, hotspots at the village-level spatial scale can serve as a reservoir of the *Plasmodium* parasite in a receptive environment, thereby sustaining and fuelling ongoing transmission or resurgences of malaria^10^ and threatening achievements made in malaria elimination settings. Fine-scale spatial targeting of malaria interventions to high-risk households or villages may therefore be a favourable approach to inform operational decisions for malaria control and elimination particularly in the context of maximising limited resources^11–13^. Moreover, a recent review has highlighted that the limited effectiveness of spatially-targeted interventions observed in several studies could be improved with better understanding of underlying drivers of ongoing transmission, better data collection and measurement of malaria hotspots^14^.

In Papua New Guinea (PNG), the scale-up of malaria interventions has resulted in a significant overall reduction in nationwide malaria prevalence from 11% in 2009 to less than 1% in 2014^15^, encouraging the realisation of its national vision to eliminate malaria by 2030^15,16^. The success of the program has been attributed to national distribution of LLINs^17^, scale-up of compulsory testing of suspected malaria cases with RDTs, and the switch to ACT for treatment^18^. Despite a standardised approach in the implementation of these measures, the reduction in malaria prevalence has not been uniform in previous high-burden areas across the country and considerable heterogeneity in malaria burden both across and within provinces has been documented^15^. The national malaria indicator survey (MIS) conducted in 2016/2017 estimated an increase in national malaria prevalence to 7.1%, with 5 out of the 18 surveyed provinces recording 8–16% prevalence and the remaining provinces 0–5%. Within lowland provinces, pockets of villages with higher prevalence were also reported^15^.

The standardised application of additional control interventions (e.g. residual spraying) using entire provinces as the implementation unit may not be cost effective if malaria risk at the district, village and household levels exhibits large degrees of heterogeneity. This may be expected given the great diversity in human behaviours and physical environments in PNG. Understanding malaria risk heterogeneity at the village level could therefore help identify geo-spatial, environmental, household, and behavioural risk factors that are perpetuating malaria transmission so that sub-Provincial micro-stratification plans can be developed, and control tools and approaches optimized. Specifically targeting appropriately identified foci of transmission with a suite of additional control tools at high coverage could accelerate transmission reduction as compared to continued uniform application of existing control interventions nationwide.

This study aims to characterise and quantify the fine-scale spatial heterogeneity of malaria infection and its changes over time in two villages in PNG, drawing upon data from two cross-sectional studies—one conducted in 2014 when national prevalence was very low, and one conducted in 2016/17 when prevalence had increased. Using a geospatial statistical modelling approach, we aimed to identify if within-village hotspots existed in 2014 and how they may have changed over this period of time as prevalence increased. In addition, we sought to identify specific risk factors that may have been associated with these hotspots.

## Methods

### Study area and data sources

**Figure 1:**
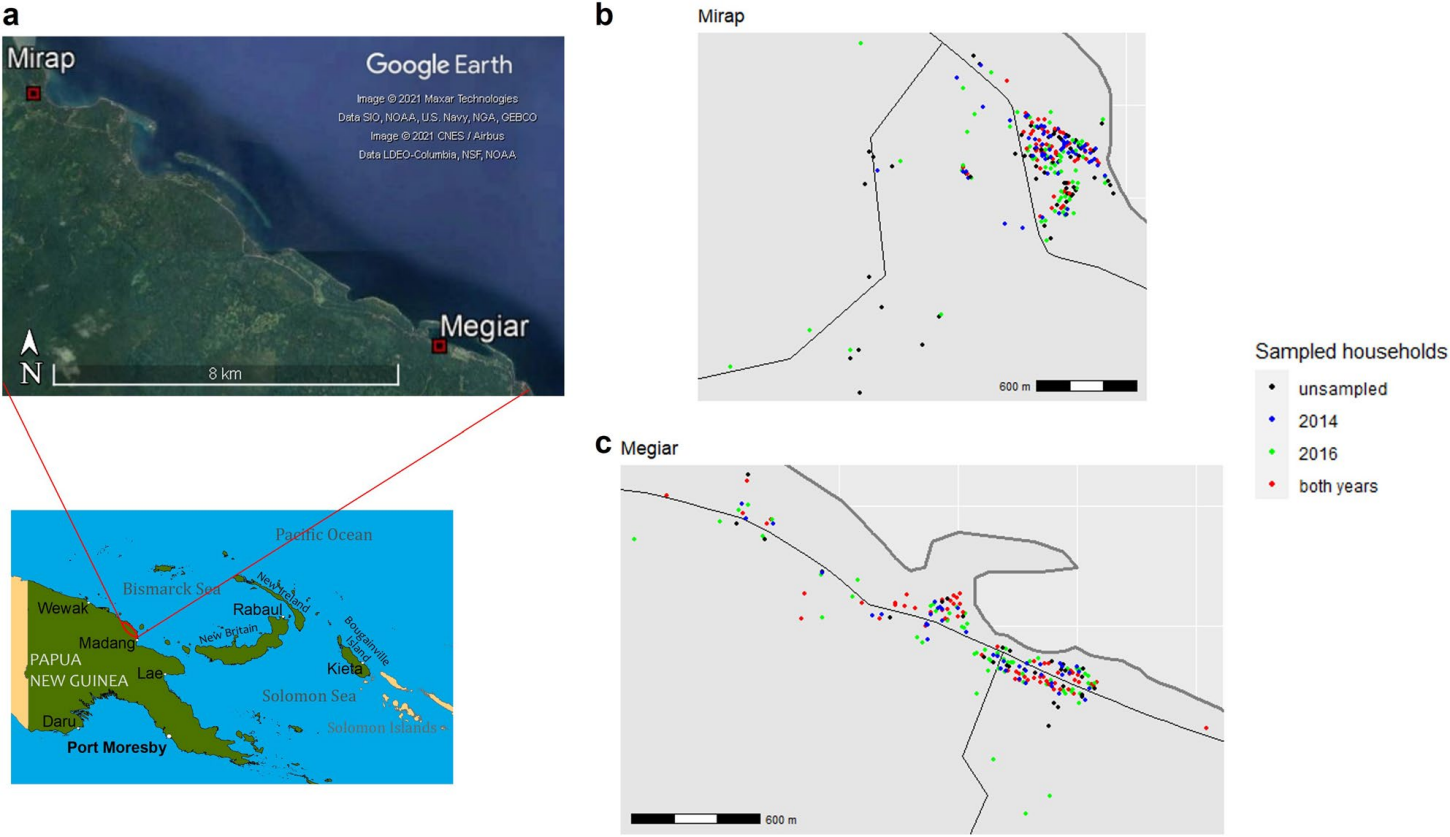
Locations of Mirap and Megiar villages in Madang Province, PNG (**a**) and spatial distribution of sampled households for community prevalence surveys conducted in 2014 and 2016 in Mirap (**b**) and Megiar (**c**). Thick grey lines denote the coastline, thin black lines denote the main road. Google Earth image from 2015. Map of PNG generated with ArcMap 10.7 (https://desktop.arcgis.com/en/arcmap/). All other maps (b and c) generated with R v.3.6.2 (https://www.R-project.org/).

Independent cross-sectional malariological surveys were conducted in PNG, Madang Province in 2014^19^ and 2016–2017 (shortened as 2016 from hereon)^20,21^. These surveys covered a number of villages on the north coast but for the purpose of this study, two villages that were included in both surveys, Megiar and Mirap, were selected to compare hotspots and risk factors between 2014 and 2016. A hotspot is defined in this study as an area with a higher than expected malaria burden were the disease to be homogeneously distributed across the study area. Geospatial analysis was an explicit aim of the 2016 survey but not of the 2014 survey. The 2014 survey utilised a convenience sampling approach, which can potentially bias participant selection. Despite this, the geographical distribution of sampled households was representative as shown in Fig. 1c. In 2016, households were randomly selected based on a village census with more than 50% sampling coverage in each village.

The two coastal villages are located 12 km apart along the north coast of Madang province, 50 km north of Madang town. Subsistence farming, fishing and trade are the main economic activities. The total geographical area of each village is approximately 4 km^2^ for Megiar and 2 km^2^ for Mirap. The households sampled in 2014 and 2016 were not the same, although 32% of households in Megiar and 21% in Mirap were common to both surveys (Fig. 1). The coverage of sampled households in 2016 also spanned a larger area than in 2014. Changes over time of hotspot location and spatial risk were analysed within each village between 2014 and 2016. No between-village comparisons were made due to the small number of villages in the study.

In both surveys, individual-level data such as demographics, history of febrile illness and LLIN usage for each participant were recorded during the finger-prick blood collection. In case of reported febrile illness or axillary temperature > 37.5 °C for an individual, a RDT (CareStart Malaria (Pf/PAN), Access Bio, USA) was performed and those positive by RDT were treated according to PNG Standard Treatment Guidelines^22^.

Geo-location of households was determined by GPS recording devices with a resolution of approximately 3–10 m in 2014 and 2016. The primary unit of analysis was the individual. In addition to the individual-level data collected from all participants, information on household characteristics such as type of construction materials used, toilet facilities, source of drinking water, income source of head of household and household agricultural assets was also collected in 2016 through questionnaires administered to the head of the household. Household-level variables were assigned to each individual residing within the household that took part in the survey.

As part of each survey, 250–300 µl of capillary blood from a finger prick sample was collected for detection of malaria parasites by light microscopy as well as by qPCR. Thick/thin blood smears were prepared on slides and examined independently by two microscopists (WHO-certified Level 1–3), with discrepancies resolved by a third microscopist (WHO-certified Level 1 Expert). Parasite densities were calculated from the number of parasites per 200 or 500 white blood cells (WBC) (depending on parasitaemia) and an assumed total peripheral WBC count of 8000 per µl, with the final density recorded as the geometric mean of the two values^23^.

For molecular detection of *Plasmodium* parasite species, DNA was extracted from 200 µl of whole blood followed by qPCR. Details of laboratory procedures were previously described for the 2014 samples^19^ and a sub-set of the 2016 samples^21^. A sub-set of the 2016 samples was extracted using NucleoMag ® Blood Beads (Macherey–Nagel, France), eluted in 200µL of MBL 5 buffer and stored in 96 well plates at − 30 °C until their use. Two duplex TaqMan qPCR assays were performed combining specific primers and probes targeting the 18 s rRNA gene region for *P. falciparum* and *P. vivax* in one reaction, and *P. malariae* and *P. ovale* in another, as reported by Rosanas-Urgell et al.^24^, with slight modifications.

### Normalised difference vegetation index (NDVI)

NDVI is a commonly used index derived from remote sensing data to measure the density of vegetation in the area. More dense vegetation is denoted by the index being closer to 1 and index closer to 0 signifies little vegetation, while negative values towards -1 denote the presence of water bodies. NDVI has been used as a proxy to infer vector density^25^, which itself has previously been reported to have a positive relationship with malaria infection risk^26^. It is therefore suitable for our models given the presence of dense vegetation surrounding households in the surveyed villages. NDVI is calculated using the standard equation:

$$NDVI= \frac{NIR-RED}{NIR+RED}$$

where NIR (near infra-red, wavelength 845–885 nm) and RED (red, wavelength 630–680 nm) are the respective spectral reflectance measurements recorded by the remote sensing satellite. Both spectral wavelengths have a spatial resolution of 30 m. Spectral data captured on 11 March 2016 from the Landsat 8 Operational Land Imager (OLI) was downloaded from the United States Geological Survey (USGS; http://earthexplorer.usgs.gov) and used as there was minimal cloud cover over our study areas on that day and we assumed that vegetation did not vary significantly over the two timepoints. The vegetation density is similar in both villages with a median NDVI value of 0.5 (Supplementary Fig. 1).

### SES index determination

Principal component analysis (PCA) was used to create a socio-economic status (SES) index from 6 factors derived from the 2016 survey data: (1) highest level of school completed by head of household, (2) main source of income of household, (3) number of chickens owned in household, (4) number of pigs owned in household, (5) number of dogs owned in household, and (6) number of cats owned in household. No autocorrelation between these factors was observed and internal validation of the SES index was determined by assessing correlation with household characteristics using the Spearman’s rank-order correlation test.

### Data analysis

Data analysis was performed with R v.3.6.2 software (R Foundation for Statistical Computing, Vienna, Austria, 2020. https://www.R-project.org/). Data from 2014 and 2016 were treated as independent data sets. Individual and household characteristics were compared between years using Chi-squared and Mann–Whitney tests. *P. falciparum* and *P. vivax* prevalence (by light microscopy and qPCR) was estimated by year, age group and village and compared between years and age groups using Chi-squared tests.

Geometric mean parasite density (expressed as PCR copy numbers/µl by qPCR) was summarised by year and village. Comparisons of parasite density between the two time points for each species in each village was performed by Wilcoxon rank test.

### Spatial and risk factor analysis

Generalised additive modelling (GAM) was used to define hotspots of malaria risk. Spatial GAM outputs are continuous surfaces of variable shape to quantify the spatially attributable risk or probability of infection across the whole study area. GAM takes a semi-parametric approach that allows for construction of an adjusted odds ratio (AOR) surface that includes the effects of covariates, and a spatially smoothed function of the residual spatial risk. Compared to a generalised linear model, which assumes all covariates have a linear relationship with the outcome, the GAM has flexibility to model complex non-linear relationships.

The GAM framework was implemented through the ‘mgcv’ package^27^ in R. The outcome of infection status for *P. falciparum* and *P. vivax* was modelled separately as a binomial response through a logit link function. Covariates chosen for the model were risk factors such as age and sex that have been epidemiologically associated with malaria infection and also other risk factors that may impact exposure to vector biting. These covariates were available in our data and were chosen based on existing evidence of their causal relationship (both direct and indirect) with malaria infection as described by a directed acyclic graph. As such we have not provided any adjustment for multiple testing and our model was an adjusted multivariate model. A base model comprising of vegetation density (NDVI) and individual-level risk factors—age, sex and LLIN use was created to estimate changes in AOR between 2014 and 2016. LLIN use was regarded as an individual behavioural risk factor modelled as a binary variable, Yes–No, in response to the prevalence survey question “did you use a bednet last night?”. A second extended model was also created using the base model covariates as well as household-level risk factors collected in the 2016 survey as covariates. This was to determine if some of the residual spatial risk predicted in the base model could be explained by household risk factors. Household level risk factors include socioeconomic status^28,29^, household construction materials for the wall and the roof^30^ and the type or lack of window screening^26,31^, type of toilet facility and source of drinking water (internal or external) were included a priori as potential malaria risk factors.

The GAM model used in our analysis was structured as follows:

$$logit\left({P}_{i}\right)=a+\sum_{n=1}^{n}{b}_{n}{x}_{ni}+c\left({SES}_{i}\right)+u\left({age}_{i}\right)+v\left({NDVI}_{i}\right)+w({L}_{i})$$

where *P* represents the probability of infection at location *i*, *a* represents the intercept, *b* represents the parametric coefficients for *n* categorical variables (*x*), *c* represents the parametric coefficient for SES (continuous variable), and *u* and *v* represents the smoothing function of a thin plate regression spline on age and vegetation density, thus accounting for the non-linear relationship between these two predictors and malaria infection. The ‘mgcv’ GAM model allows us to simultaneously estimate from the data all possible values for the smoothing parameters and obtain the optimal smoothing parameter by maximizing the penalized likelihood function. For this purpose, we have set the smoothing parameter estimation method attribute in the model to the restricted maximum likelihood (REML) method. Preliminary modelling showed that SES had a linear relationship and therefore was modelled using a simple parametric term. The spatial effect of household location *L* (longitude and latitude coordinates) was modelled as an interactive term through the thin plate regression spline function, *w*, with basis function following a Gaussian process. The mathematical formulation for the base model is similar to the extended model but with the removal of the *c(SES*_*i*_*)* term and a reduced matrix of *n* categorical variables to individual level factors of sex and LLIN use. We used empirical variograms to investigate the model residuals to ensure that there was no further household spatial dependency and autocorrelation in our models.

The GAM was then used to predict the adjusted log odds for malaria infection over a continuous space across the study area. To accomplish this, a prediction grid using latitude and longitude with a resolution of 0.0001 degrees was created, encompassing the study area. Each grid intersection point therefore formed a location for model prediction. To complete the covariates for prediction, we created an ‘average’ model by taking the mode and median values for categorical and continuous covariates respectively so that the variation of the predicted log odds was due to its spatial location in the study area. The NDVI covariate values were projected on the prediction grid to capture the actual variation of vegetation across the study area. A ‘null’ model was also created by omitting all covariates to provide a reference for average odds of infection over the entire study area (equivalent to the ratio of cases to controls). This reference was then used to calculate the AOR of spatial effects at each location. A hotspot, as predicted from the GAM model, is therefore defined as an area with AOR greater than 1, representing locations where malaria risk was higher than what would be expected were the disease to be homogeneously distributed across the study area. Covariate effects were expressed as AORs.

### Ethics approval

Study protocols for both surveys were approved by the Institutional Review Board of the PNG Institute of Medical Research (IRB 1116 & 1517), the PNG National Medical Research Advisory Committee (MRAC 11.21 & 16.08), Alfred Precinct (Project No. 721/18) and Monash University (Project No. 18151). Written informed consent was obtained from all study participants or their parents or legal guardians and research was conducted in accordance with the Declaration of Helsinki.

## Results

### Study population and summary

In Megiar, 509 individuals living in 121 households were included in the 2014 survey and 601 individuals in 132 households were included in the 2016 survey. In Mirap, 529 individuals living in 120 households and 672 individuals living in 127 households were included in 2014 and 2016 respectively.

There were no statistically significant differences between sex and age distributions of study participants in both years. LLIN usage in both years in both villages was > 80% (Supplementary Table 1). A summary of household characteristics collected in the 2016 survey is presented in Supplementary Table 2.

### Prevalence of infections in 2014 and 2016

The prevalence of qPCR-detected *P. falciparum* increased between 2014 and 2016 in each village (Megiar: 11.4 to 19.1%, p < 0.001; Mirap: 12.3 to 28.3%, p < 0.001). By contrast, the prevalence of *P. vivax* infection was similar over the two years (Megiar: 20.6 to 18.3%, p = 0.33; Mirap: 22.1 to 23.4%, p = 0.61). *P. vivax* was dominant in 2014 with a higher prevalence than *P. falciparum*. However, in 2016, *P. falciparum* was the dominant species, particularly in Mirap. The prevalence of malaria infection as detected by light microscopy (LM) and qPCR for each village at each of the two time points is summarised in Table 1.

**Table 1:** Prevalence of *Plasmodium* spp. infection by light microscopy and polymerase chain reaction in Megiar and Mirap in 2014 and 2016.

	Megiar			Mirap		
	2014 (%)	2016 (%)	p-value^a^	2014 (%)	2016 (%)	p-value^a^
**Species LM prevalence**			< 0.001			< 0.001
Number tested	509	601		529	671^b^	
Pf	9 (1.8)	42 (7.0)		12 (2.3)	68 (10.1)	
Pv	15 (3.0)	32 (5.3)		20 (3.8)	60 (8.9)	
Pm	0	0		0	1 (0.2)	
Mixed	1 (0.2)	8 (1.3)		7 (1.3)	2 (0.3)	
**Species qPCR prevalence**			0.002			< 0.001
Number tested	509	600^b^		529	670^b^	
Pf	46 (9.0)	88 (14.7)		49 (9.3)	146 (21.8)	
Pv	94 (18.5)	82 (13.7)		104 (19.7)	119 (17.8)	
Pm	4 (0.8)	8 (1.3)		4 (0.8)	2 (0.3)	
Po	0	2 (0.3)		0	1 (0.2)	
Mixed	11 (2.2)	27 (4.5)		11 (2.1)	38 (5.7)	
All malaria^c^ positive^d^	156 (30.7)	209 (34.8)	0.145	170 (32.1)	312 (46.4)	< 0.001
Pf^e^ positive	58 (11.4)	115 (19.1)	< 0.001	65 (12.3)	190 (28.3)	< 0.001
% Pf sub-microscopic^f^	80.4	52.3		75.5	53.4	
Pv^g^ positive	105 (20.6)	110 (18.3)	0.329	117 (22.1)	157 (23.4)	0.609
% Pv sub-microscopic^f^	84.0	61.0		80.8	49.6	

*LM* light-microscopy detected, *qPCR* PCR detected, *Pf* *P. falciparum*, *Pv* *P. vivax*, *Pm* *P. malariae*, *Po* *P. ovale.*

^a^p-value based on chi-square test comparing prevalence between 2014 and 2016 within each village.

^b^Number tested differ from total sample size at timepoint in village due to missing values.

^c^Tested positive for malaria infection (includes all species).

^d^Indicate positive infection as detected either by light microscopy or by qPCR. Denominators used are same as village sample size at that time point.

^e^Single *P. falciparum* infection and mixed species infection that includes *P. falciparum.*

^f^Proportion of infections that were detected by qPCR only and not by light microscopy.

^g^Single *P. vivax* infection and mixed species infection that includes *P. vivax.*

**Figure 2:**
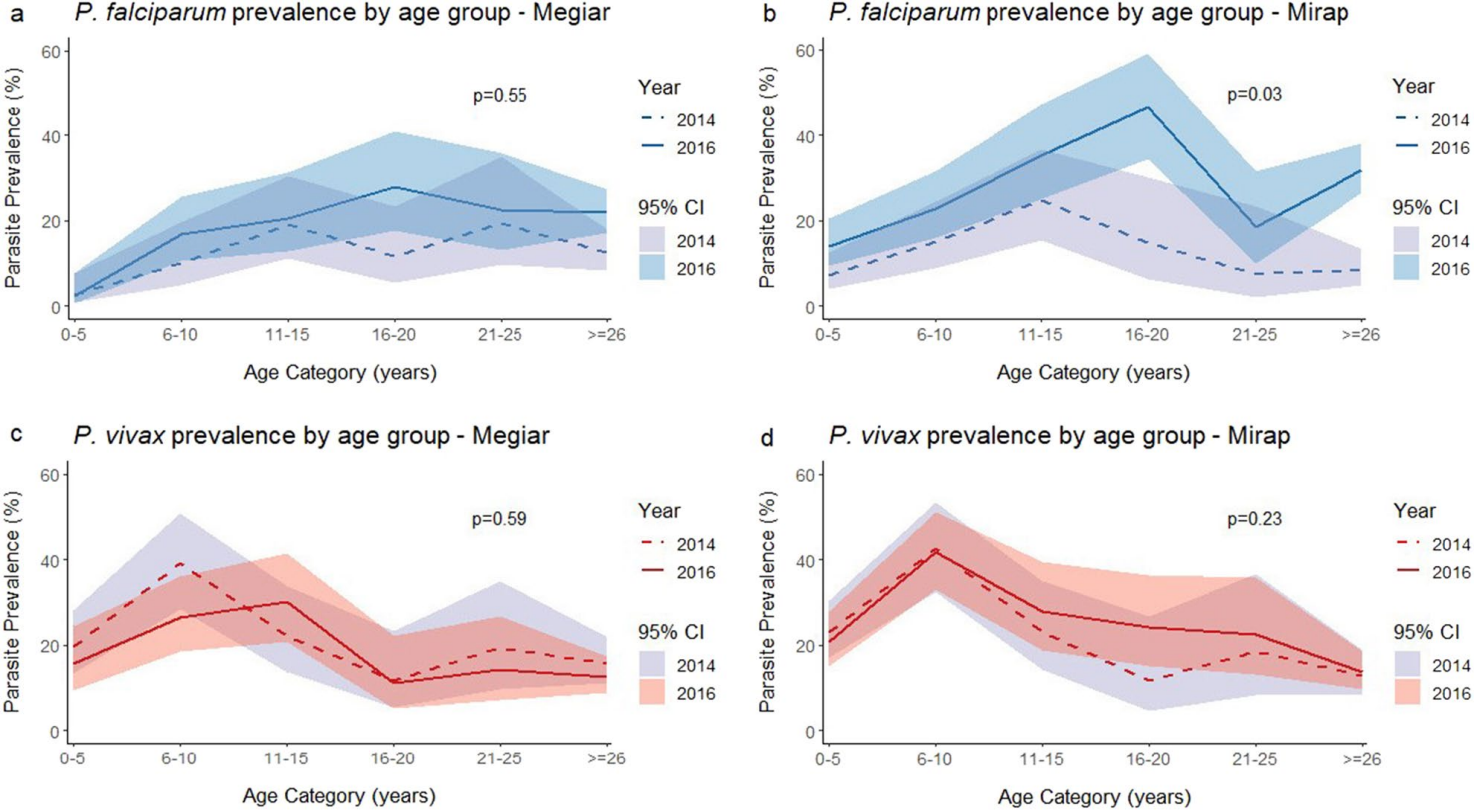
Malaria infection prevalence by age group and year for *P. falciparum* in Megiar (**a**), *P. falciparum* in Mirap (**b**), *P. vivax* in Megiar (**c**), and *P. vivax* in Mirap (**d**). 95% confidence interval (CI) denoted by coloured bands. P-values denote the test of independence of prevalence trend by age groups between 2014 and 2016 in each village.

Comparing age trends in malaria prevalence, the prevalence of *P. falciparum* and *P. vivax* by qPCR in both villages was observed to increase with age. *P. falciparum* prevalence reached its peak in the 11–15 years age group in 2014 and in the 16–20 years age group in 2016 (Fig. 2a,b), while the peak for *P. vivax* was in younger ages, mostly in children aged 6–10 years (Fig. 2c,d). The observed difference in prevalence trend between years was only statistically significant for *P. falciparum* in Mirap (Fig. 2b; p = 0.03).

### Parasite density of infections in 2014 and 2016

For both *P. falciparum* and *P. vivax*, an increase in parasite density and a decrease in the proportion of sub-microscopic infections was observed between 2014 and 2016, indicative of higher transmission. The geometric mean parasite density by qPCR was higher in 2016 than in 2014 for both species in Megiar and Mirap. In Megiar, geometric mean for *P. vivax* was 1.6 copies *P. vivax* 18S rDNA/µl (95% CI 3.4, 7.2) in 2014 and 84.6 copies/µl (95% CI 52.2, 137.1) in 2016, while for *P. falciparum*, parasite density was 52.6 copies/µl (95% CI 26.8, 103.3) in 2014 and 431.7 copies/µl (95% CI 215.8, 863.8). In Mirap, geometric mean for *P. vivax* was 29.7 copies/µl (95% CI 19.5, 45.2) in 2014 and 71.6 copies/µl (95% CI 45.3, 113.1) in 2016, while for *P. falciparum*, parasite density was 117.3 copies/µl (95% CI 57.7, 238.7) in 2014 and 183.8 copies/µl (95% CI 104.1, 324.6) in 2016. These differences between years were statistically significant except for *P. falciparum* in Mirap (Supplementary Fig. 2). In both years, a considerable proportion of infections were sub-microscopic (or low-density) for both species, although the proportion of low-density infections was lower in 2016 when overall prevalence had increased (Megiar, 2014 to 2016: 80.4 to 52.3% for *P. falciparum*, 84.0 to 61.0% for *P. vivax;* Mirap, 2014 to 2016: 75.5 to 53.4% for *P. falciparum*, 80.8 to 49.6% for *P. vivax*) (Table 1).

### Risk factor analysis

Age was a consistent risk factor for qPCR-detected *P. falciparum* and *P. vivax* infections, in both villages and at both time-points (range p < 0.001 to p = 0.05), except for *P. falciparum* in Mirap in 2014 (p = 0.09), as shown in our models. The effect of age on probability of infection is shown as smoothed partial effect plots in Supplementary Fig. 3. Generally, probability of infection increased as age increased to 10 to 15 years of age for *P. vivax* (Supplementary Fig. 3b, d, f, g) and *P. falciparum* (Supplementary Fig. 3e, g) respectively. However, in Megiar the effect of age on *P. falciparum* infection continued to increase to 20 years of age (Supplementary Fig. 3a, c). Infection probability started to decline as age increased into adulthood. The confidence intervals also became wider for older age groups due to their smaller sample size.

In Megiar in 2016, males had a significantly higher odds of *P. falciparum* infection compared to females (AOR = 1.58, 95% CI 1.02–2.46), whereas sex was not a significant risk factor in Mirap (Table 2). LLIN usage was associated with reduced odds of *P. vivax* infection in Megiar (AOR = 0.50, 95% CI 0.26–0.96, p < 0.05) but was not associated with risk of *P. falciparum* infection.

**Table 2:** Individual and household risk factors for individual *Plasmodium * spp. infection by village in 2016.

Parametric covariates	Megiar		Mirap	
	*P. falciparum*	*P. vivax*	*P. falciparum*	*P. vivax*
**Sex–Male**	1.58**	1.14	1.15	0.96
	(1.02–2.46)	(0.72–1.82)	(0.80–1.66)	(0.65–1.41)
**Bednet usage**	1.07	0.50**	0.96	0.62
	(0.58–2.00)	(0.26–0.96)	(0.45–2.08)	(0.26–1.48)
**Windows**				
All screened	Ref	Ref	Ref	Ref
No windows /not screened	1.11	1.27	1.35	0.50*
	(0.61–2.04)	(0.64–2.49)	(0.67–2.74)	(0.26–1.00)
Partly screened	0.80	1.08	0.95	0.66
	(0.47–1.37)	(0.61–1.94)	(0.45–2.01)	(0.32–1.33)
**Toilet facility**				
Own pit latrine/flushed toilet	Ref	Ref	Ref	Ref
No facility/bush/seashore	1.00	0.81	0.40**	1.84
	(0.53–1.90)	(0.40–1.61)	(0.20–0.82)	(0.77–4.41)
Shared pit latrine/flushed toilet	0.88	0.55*	0.57	2.47
	(0.50–1.54)	(0.29–1.04)	(0.12–2.75)	(0.44–13.80)
**Drinking water source**				
Water tank/Piped into dwelling	Ref	Ref	Ref	Ref
Surface water (river, pond, etc.)	1.72*	2.10**	0.48	1.12
	(0.88–3.37)	(1.01–4.39)	(0.15–1.55)	(0.30–4.23)
Well (open/protected; public/private)	NA	NA	0.71	1.79
			(0.33–1.53)	(0.73–4.39)
Piped into neighbourhood/public tap	1.58	1.98*	0.98	1.17
	(0.87–2.87)	(0.97–4.01)	(0.22–4.28)	(0.20–6.89)
**Wall material**				
Cement/bricks/iron sheets	Ref	Ref	Ref	Ref
Bamboo/pitpit	2.64	5.70*	1.11	NA
	(0.63–11.00)	(0.95–34.20)	(0.34–3.62)	
Sago leaf stalks	2.18	3.65	1.50	NA
	(0.53–9.02)	(0.62–21.40)	(0.48–4.71)	
Wood/Mansonite/Fibrowood	1.60	5.14*	0.63	NA
	(0.37–6.93)	(0.84–31.30)	(0.09–4.36)	
**Roof material**				
Corrugated iron	Ref	Ref	Ref	Ref
Thatched grass/Sago palm leaves	0.53**	0.63	1.02	0.87
	(0.31–0.92)	(0.35–1.15)	(0.49–2.10)	(0.44–1.71)
**SES index**	1.03	0.97	0.84	0.92
	(0.88–1.20)	(0.82–1.16)	(0.71–1.00)	(0.77–1.10)

Adjusted odds ratios from GAM. Data in parenthesis denote 95% confidence interval.

Symbols and abbreviations: *** p < 0.01; ** p < 0.05; * p < 0.1; Pf, *P. falciparum*; Pv, *P. vivax*; Ref, reference strata; NA, not applicable as characteristic was not present in village or value was not computable; SES, Socio-economic status.

These odds ratios are also age-adjusted and vegetation density-adjusted. Non-parametric smooth effects of age and vegetation index are shown as graphs in Supplementary Fig. 3 and 4 respectively.

Specific household factors were observed to be associated with higher odds of malaria infection in Megiar. Compared to households that had piped water into the house or had a water tank in their compound, households who used surface water were at a higher risk of *P. vivax* infection (Megiar: AOR = 2.1, 95% CI 1.01–4.39, p < 0.05) (Table 2). Interestingly roofs that were constructed from traditional thatched grass or sago palm leaves were associated with reduced odds of *P. falciparum* infection compared to more modern corrugated iron roofs in Megiar (AOR = 0.53, 95% CI 0.31–0.92, p < 0.01). In contrast, traditional materials such as bamboo or sago leaf stalks that were used to construct the walls of houses were associated with increased odds of infection compared to modern materials such as cement, bricks, or iron sheets (AOR range = 1.6–5.7), but these estimates were not statistically significant and the wide confidence interval is indicative of sparse data available (Table 2). In Mirap, the majority of household risk factors examined had AOR close to 1 and were not significant (Table 2), suggesting household factors in Mirap may be less variable and/or less influential than in Megiar.

Overall, denser vegetation (an increased NDVI) was generally observed to have a weak positive non-significant relationship with malaria infection in both villages (Supplementary Fig. 4a–h). However, in Mirap in 2016, risk of *P. falciparum* infection was significantly and positively associated with NDVI (p < 0.01) (Supplementary Fig. 4i, k). Conversely, a negative relationship for *P. vivax* was observed in Mirap in 2016 (Supplementary Fig. 4j, l), indicative of an increased risk of vivax infection in less densely vegetated areas of the village predominated by houses and community buildings.

### Spatial analysis

**Figure 3:**
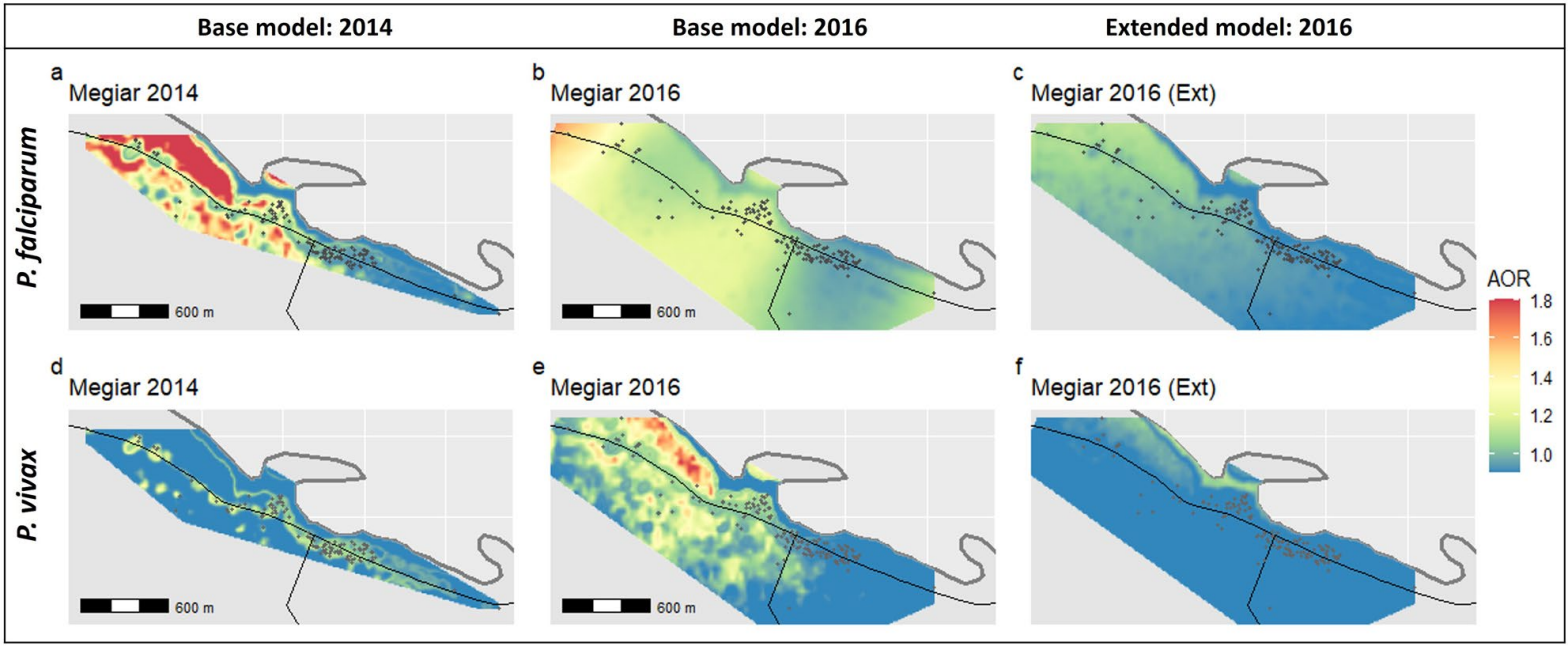
Variable spatial effect of predicted adjusted odds ratios (AOR) for *P. falciparum* and *P. vivax* infection derived from generalised additive models (GAM) in Megiar. Base model covariates age, sex, LLIN usage and NDVI were applied in the GAM for 2014 and 2016 (**a**,**b**,**d**,**e**). Base model covariates combined with household covariates (extended model) were applied in the GAM in 2016 (**c**,**f**) to investigate the contribution of household risk factors to spatial risk. Spatial distribution of sampled households shown in grey points in both the 2014 and 2016 base models. Megiar is a coastal village, thick grey lines represent the coastline and thin black lines represent the main road. Maps were created in R v.3.6.2 (https://www.R-project.org/).

In Megiar, spatial heterogeneity for *P. falciparum* was observed in 2014 with higher odds of infection predicted for focal areas in the northern part of the study area (Fig. 3a, Supplementary Fig. 5a–c). When the prevalence of *P. falciparum* increased in 2016, these focal hotspots expanded and merged with overall AOR values ranging between 1.1–1.4 (Fig. 3b, Supplementary Fig. 6a–c). These areas were considered statistically significant hotpots with the lower bound 95% confidence interval AOR > 1 (Supplementary Fig. 6a). It should be noted that high estimated OR on the edges of the study area is likely to be influenced by edge effects and sparse observations at the margins of the study area. When household covariates were incorporated into the extended model, spatial risk for *P. falciparum* in Megiar became almost homogenously distributed, suggesting that the residual spatial risk observed in 2016 was largely due to household risk factors (Fig. 3c, Supplementary Fig. 7a–c).

In 2014, limited spatial risk for *P. vivax* was observed and restricted to those areas of the village where houses were located along the road (Fig. 3d). In 2016, spatial risk was more marked in the northern part of the study area with AOR values ranging between 1.1–1.6 as predicted under the base model (Fig. 3e). Although hotspots for *P. vivax* were identified in both years, only small sections of the hotspots were observed to be statistically significant (based on 95% confidence interval) in both years, although more evident in 2016 (Supplementary Fig. 5d–f, 6d–f). The AOR surface became almost homogeneous with an AOR of 1 in the extended model (Fig. 3f), again suggesting that household risk factors could largely explain the residual spatial risk observed in Megiar when transmission increased.

**Figure 4:**
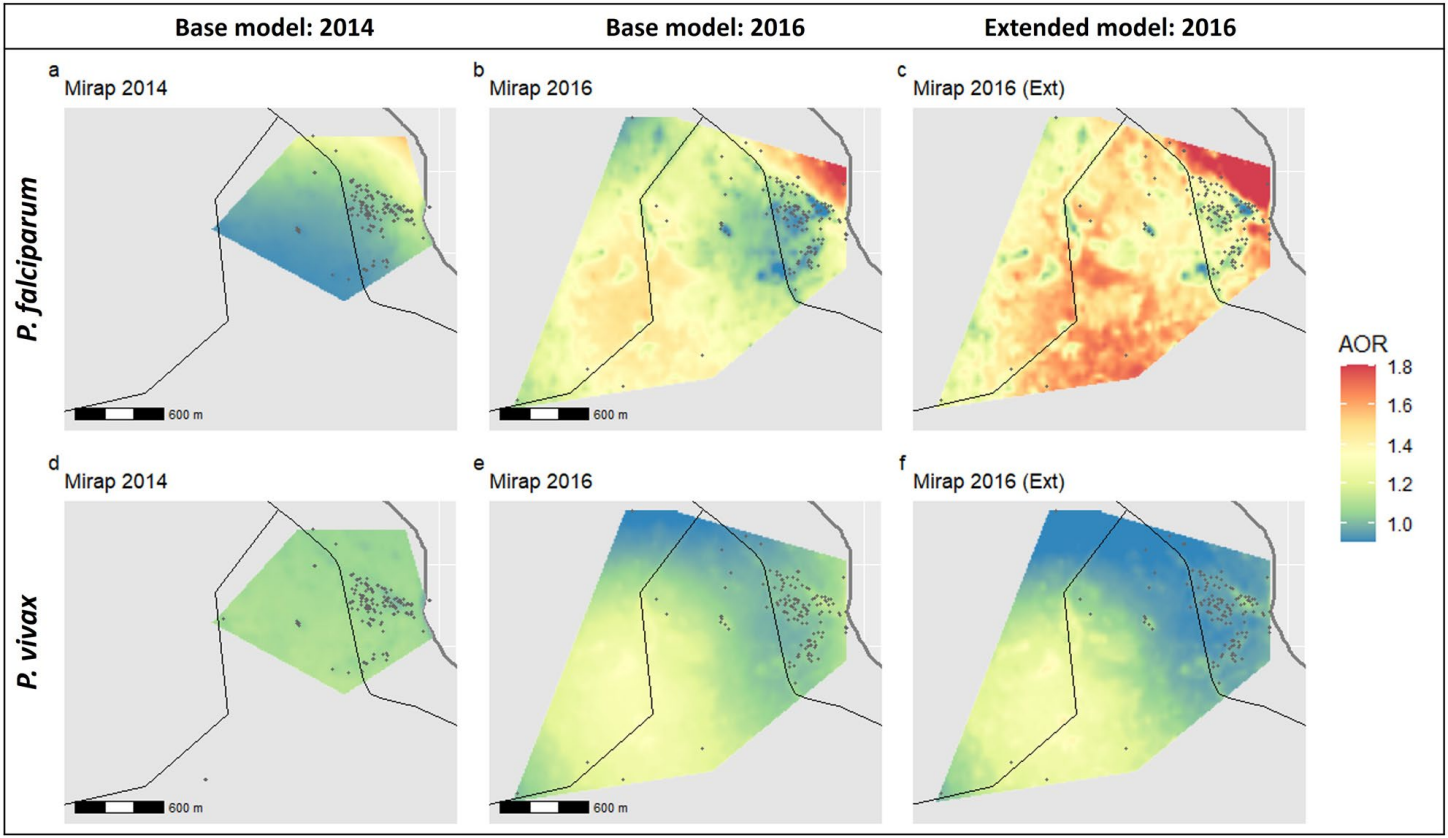
Variable spatial effect of predicted adjusted odds ratios (AOR) for *P. falciparum* and *P. vivax* infection derived from generalised additive models (GAM) in Mirap. Base model covariates age, sex, LLIN usage and NDVI were applied in the GAM for 2014 and 2016 (**a**,**b**,**d**,**e**). Base model covariates combined with household covariates (extended model) were applied in the GAM in 2016 (**c**,**f**) to investigate the contribution of household risk factors to spatial risk. Spatial distribution of sampled households shown in grey points in both the 2014 and 2016 base models. Mirap is a coastal village, thick grey lines represent the coastline and thin black lines represent the main road. Maps were created in R v.3.6.2 (https://www.R-project.org/).

In Mirap, the AOR for *P. falciparum* and *P. vivax* infection was homogenously distributed across the study area in 2014 (Fig. 4a,d). When prevalence for *P. falciparum* and *P. vivax* increased in 2016, an area in the south-west of the village was observed to have a higher *P. falciparum* AOR > 1.3 (Fig. 4b) and *P. vivax* AOR between 1.2–1.3 (Fig. 4e). It is important to note that this particular hotspot area was not part of the village area that was sampled in the 2014 survey and so comparison of AOR between 2014 and 2016 in this particular area of Mirap is not possible. In contrast to this hotspot area in the south-west of the village, the area in the north-western part of the village towards the coastline, where most of the houses in Mirap are located, AOR was much lower between < 1.0–1.1. Comparing the spatial risk in this area between 2014 and 2016 revealed that the AOR estimate did not substantially change.

Incorporating household covariates in the extended model for 2016 did not significantly change the AOR for *P. vivax* suggesting that residual spatial risk could not be explained by household factors in this case (Fig. 4f). However, the AOR increased overall across the village for *P. falciparum* in the 2016 extended model (Fig. 4c). It is not clear why this is so, although both the base and extended model revealed a significant contribution by vegetation density (p = 0.005 and p = 0.001 respectively) (Supplementary Fig. 4i, k) towards spatial risk.

The hotspot observed in 2016 was associated with increased vegetation density suggesting increased transmission risk of both *P. falciparum* and *P. vivax* due to increased likelihood of host-vector contact. However, we caution that this risk prediction in the 2016 hotspot may be biased and over-inflated due to the sparse sampling of households in this area. The lack of precision of the AOR estimate is inferred from the wider confidence intervals in this hotspot as compared to the other areas of Mirap (Supplementary Fig. 10).

## Discussion

Understanding the extent of village-level spatial heterogeneity of malaria risk is important for malaria control and elimination programs to determine if sub-Provincial spatial targeting to high-risk areas is worthy of consideration. This involves quantifying spatial risk through identifying hotspot areas as well as uncovering specific risk factors that may be driving increased risk of infection in these areas. In this study, we specifically explored if hotspots of malaria risk existed in two villages on the north coast of PNG, how hotspots varied over a period when increased malaria transmission was observed despite intensive malaria control in the years before, and if any identified risk factors could explain the differential risk between the two villages.

Following a period of rapidly declining *P. falciparum* and *P. vivax* prevalence and density of infections, from 2006 to 2014^19^, the prevalence in 2016 had increased in both villages. For both species, an increase in parasite density and a decrease in the proportion of low density (sub-microscopic) infections was observed, indicative of higher transmission. *P. falciparum* and *P. vivax* hotspots were observed in these village and their location, size and intensity differed according to village-specific characteristics associated with transmission. Hotspots for *P. falciparum* were more clearly observed than for *P. vivax*.

Spatial risk heterogeneity for *P. falciparum* was observed in Megiar in both years, even though prevalence of infection and parasite density increased between 2014 and 2016. The hotspots increased in size between 2014 and 2016 and an upward shift in the age-specific peak prevalence to the 16–20 years age-group was also observed. Spatial risk heterogeneity was observed for *P. vivax* in Megiar, but risk was more focal. Although *P. vivax* prevalence was similar in both years, the density of infections increased significantly in 2016 and an upward shift in the peak prevalence to the 11–15 years age-group was observed. Much of the spatial risk for both species in Megiar could be explained by household risk factors. In particular, the use of surface water as a drinking water source and use of corrugated iron roofing, both of which are more likely to increase the risk of vector exposure.

In contrast, spatial risk for *P. falciparum* and *P. vivax* was homogeneous in the area of Mirap that was sampled in both years. It is possible that in this highly populated village, transmission had not yet declined to a level in 2014 where hotspots could be observed. *P. falciparum* prevalence increased significantly in 2016 and peak prevalence shifted to the 16–20 years age group, but the mean density of infections was similar. *P. vivax* prevalence was similar in both years, but parasite density increased significantly in 2016. Household factors did not explain the residual spatial risk, instead a hotspot was predicted for the sparsely populated south-west area with dense vegetation, which showed a significant positive association with infection risk.

In Megiar, there was evidence that 2014 hotspots may have expanded by 2016, supporting observations from other settings that hotspots fuel ongoing transmission if neglected^32,33^. It is possible that the 2014 hotspots acted as a reservoir of infections at a time when LLINs with reduced bioefficacy^34^ were distributed in the area in 2015^35^, thereby fuelling the expanded hotspot in 2016. Entomological data from the area also showed that infective biting rates were observed to increase by threefold in Megiar and 18-fold in Mirap between 2010 and 2016 and that the majority of vectors were caught outdoors (personal communication, J. Keven). Differences in vector competence between *P. falciparum* and *P. vivax* in PNG has been reported^36^ and may also be a factor underlying hotspot variation observed in our study.

The hotspot observed in Mirap in 2016 warrants careful interpretation, notably because the density of houses in this area is low, which may have biased the model risk estimates. We do however note that this hotspot coincides with a densely vegetated area of the village and such environments could be conducive for vector breeding and activity^37^ and this may have increased the risk for people living in or frequenting this area. Similarly, the observed ‘neutral spot’ that has a lower AOR for *P. falciparum* infection, is a cleared and more populated area of the village, consisting largely of the main road, community buildings and considerably less vegetation and forest. The contrast between model-predicted low (uniform) AOR in an area where the major proportion of *P. falciparum* infections and households are found, to model-predicted high AOR in an area where a small proportion of the village-specific *P. falciparum* prevalence is located, suggests that malaria transmission could be predominantly occurring in these vegetated non-habited areas where village residents frequently walk through^20^, increasing the opportunities for vector exposure.

The observed pattern for *P. vivax* prevalence is quite consistent with other co-endemic settings where in the face of intensive malaria control to reduce transmission and disease burden, *P. falciparum* prevalence decreased but *P. vivax* prevalence remained relatively stable or even increased due to relapses^38^. The homogenous nature of the observed spatial risk for *P. vivax* is likely due to the unique biology of *P. vivax,* whereby infections are due to a combination of newly transmitted infections and relapsing infections from dormant liver stage hypnozoites. In PNG, relapsing infections can occur frequently and account for up to 80% of *P. vivax* infections^39^. Frequent occurrences of *P. vivax* relapses, which are not dependent on vector exposure, may therefore have overshadowed and either focalised or homogenised the detection of any *P. vivax* ‘transmission hotspots’ in our models. New mosquito-derived *P. vivax* infections are obviously dependent on vector exposure, but importantly human to mosquito transmission may stem from either newly infected or relapsed individuals. It is therefore important to consider if ‘infection’ hotspots are also areas where there may be intense vector activity and/or greater opportunities for human-vector interaction.

At the individual level, age remained a significant risk factor with probability of infection for *P. falciparum* peaking in 15 to 20 years old adolescents and *P. vivax* in 10 years old children, which translated to highest parasite prevalence in those ages as well. This is consistent with other studies where peak prevalence and odds ratios of *P. vivax* were observed in younger children (6–12 years of age) than those for *P. falciparum* (12–20 years of age)^19,40^. Behavioural data observed from this village, confirmed that school-aged children walked to school and the further they lived away from school, the higher their potential for vector exposure as they tended to wake up earlier before dawn^20^. Our data suggests males are at higher risk, which may be explained by the lack of protective clothing for boys and men who tend to move about in shorts, short-sleeved shirts or even shirtless from before dawn to well after dusk^20^. Men have also been reported to use LLINs less often than women^20,41^.

Although an increase in prevalence was observed between 2014 and 2016, self-reported LLIN usage remained high at 82% in Megiar and increased from 85.6 to 93.9% in Mirap. This is consistent with studies showing that the effectiveness of high-coverage LLIN usage plateaued in further reducing human-vector contact and malaria prevalence^17,42–44^. LLINs have also been shown to have no further preventive effect in malaria reduction following a rebound in malaria prevalence after a long period of decline^45^. This could suggest that vector biting was occurring at earlier times when individuals were not sleeping and therefore not under LLINs or biting outdoors as previously documented in PNG^46,47^ and other settings^48–50^. Additionally, the reduced bio-efficacy of LLINs distributed in PNG since 2013^34^ is likely to be a major contributing factor towards the lack of protective effect of LLIN when the third round of LLIN distribution occurred in this area in 2015^35^. This coincides with the resurgence observed in the surveyed villages in 2016.

Incorporating household characteristics into the model explained residual spatial risk observed in Megiar but not in Mirap. The importance of household-level over individual-level factors in predicting malaria risk has been shown in other settings such as Cambodia^51^. In our study, households needing to access surface water serve as a proxy risk factor for vector exposure in this setting. The likely need to venture into dense areas of garden/forest to access the nearby river or stream for water collection places the individual at higher risk of mosquito exposure. While it may be argued that water collection and other such activities would tend to occur at dawn, dusk, or daytime rather than at night when peak vector biting rates are expected, recent studies in PNG and elsewhere have reported a shift in vector biting behaviour to feeding outdoors in the earlier hours of the evening, at a time when individuals were still outdoors and not protected by LLINs, as well as at early dawn^46,47,49,50,52^. This is supported by behavioural studies conducted in this region of PNG, highlighting water-related household chores taking place in the early morning and evening, often performed by school-aged children and likely increasing vector exposure^20^. In Mirap, only a small percentage of households (8%) used surface water as their drinking water source with most households (79%) having access to nearby wells. Use of wells was not a significant risk factor in our models, possibly related to them often being located within a village in close proximity to houses. No households indicated use of wells in Megiar, highlighting a distinct difference in this behaviour between the two villages.

House design is also known to be an important determinant of malaria risk^31^ and in some settings, specific household modifications have demonstrated efficacy as additional tools alongside LLINs and IRS^30,53^. In this study, roofs made from thatched grass or sago palm leaves were associated with up to two-fold reduced odds of malaria infection compared to corrugated iron roofs, suggesting that in this context traditional materials or the style of construction used with traditional materials may reduce vector entry. It may be that houses with corrugated iron roofs were built with openings or with open eaves in order to improve ventilation and help cool the houses^30^, but in the process has increased opportunity for vector entry as shown in settings such as in Kenya and Tanzania^42,54^. Houses with corrugated iron roofs can also be hot at night which may lead to people tending not to use LLINs when they sleep^55^. Further investigation and finer classification of roof construction as closed/intact and -open/with open eaves will be useful to further elucidate the relationship between roof as a proxy risk factor of vector exposure and malaria infection. Additional tools such as eave ribbons (containing passive mosquito repellent such as transfluthrin)^56^ and eave tubes (containing insecticide-treated discs)^57^ may also warrant further investigation for effectiveness in the PNG setting.

Our study has several limitations. Firstly, the two surveys are independent with less than 32% of participants involved in both surveys and the sampling strategy was also different. Sparse sampling in certain areas of the village may also have resulted in biased overestimated prediction of malaria risk as observed in Mirap in 2016. The 2014 survey did not collect detailed data on household characteristics, preventing investigation of household risk factors that may have been sustained throughout the two timepoints. Self-reported LLIN use may be overestimated due to recall and courtesy bias. Additional studies could extend this analytic framework to include other data such as entomological activity, and frequency of residents’ movement within village such as to rivers and vegetated areas. Furthermore, cohort studies with molecular force of infection data and/or spatially explicit health facility surveillance conducted over several years may provide additional evidence to advance our understanding of village-level spatio-temporal dynamics of transmission.

This study used a GAM approach at the village level to identify hotspots with higher spatial risk after adjusting for a range of known risk factors. Different spatial, environmental, and household risk factors were identified in two villages despite their close proximity to each other. Collecting surface water for drinking water was a household risk factor in Megiar and being surrounded by dense vegetation was a risk factor in Mirap. In both cases, these risk factors are proxies for vector exposure outdoors as villagers move about in their surroundings, suggesting a role for the application of tailored approaches targeting outdoor transmission. These results provide a rationale for consideration of the utility of sub-Provincial spatial risk mapping to identify key drivers of ongoing transmission and guide the application of additional malaria control interventions to further accelerate transmission reduction. To improve the effectiveness of interventions aiming to interrupt persistent ongoing transmission, new approaches, tools, and indicators that can better predict spatial risk and identify stubborn hotspots are required.

## Supplementary Information

Supplementary Information.

## Data Availability

The datasets generated and analysed during the current study are not publicly available due to issues of confidentiality as stipulated by the human ethics committee. Data may be available on request from the corresponding author after approval from PNGIMR to ensure they meet PNGIMR IRB and PNG MRAC requirements for access to data.
