# Supplementary material for: Investigating differences in village-level heterogeneity of malaria infection and household risk factors in Papua New Guinea
Source: Sci Rep. 2021 Aug 16;11:16540. doi: 10.1038/s41598-021-95959-8 (PMC8367982; doi:10.1038/s41598-021-95959-8)
Supplement: Supplementary file 1 — Supplementary Information. [file 41598_2021_95959_MOESM1_ESM.pdf]

Investigating differences in village-level heterogeneity of malaria infection and household risk factors in Papua New Guinea

Desmond Gul[1, 2], Daniela Rodríguez-Rodríguez[3, 4], Elma Nate[5], Alma Auwan[5], Mary Salib[5], Lina Lorry[5], John B. Keven[5,6], Michelle Katusele[5], Jason Rosado[7, 8], Natalie Hofmann[3, 4], Maria Ome-Kaius[5], Cristian Koepfli[9, 10], Ingrid Felger[3, 4], James W. Kazura[11], Manuel W. Hetzel[3, 4], Ivo Mueller[10], Stephan Karl[12], Archie C. A. Clements[13, 14], Freya J. I. Fowkes[1, 2, 15], Moses Laman[5], Leanne J. Robinson[1, 2, 5, 10*]

[1] Burnet Institute, Melbourne, Australia
[2] School of Public Health and Preventive Medicine, Monash University, Melbourne, Australia
[3] Swiss Tropical and Public Health Institute, Basel, Switzerland
[4] University of Basel, Basel, Switzerland
[5] Papua New Guinea Institute of Medical Research, Madang, Papua New Guinea
[6] Department of Microbiology and Molecular Genetics, Michigan State University, Michigan, USA
[7] Unit of Malaria: Parasites and hosts, Institut Pasteur, Paris, France
[8] Sorbonne University, Paris, France
[9] University of Notre Dame, Notre Dame, Indiana, USA
[10] Walter and Eliza Hall Institute of Medical Research, Melbourne, Australia
[11] Case Western Reserve University, Cleveland, Ohio, USA
[12] James Cook University, Cairns, Australia
[13] Curtin University, Perth, Australia
[14] Telethon Kids Institute, Perth, Australia
[15] Melbourne University, Melbourne, Australia
* (email: leanne.robinson@burnet.edu.au)

Supplementary Material

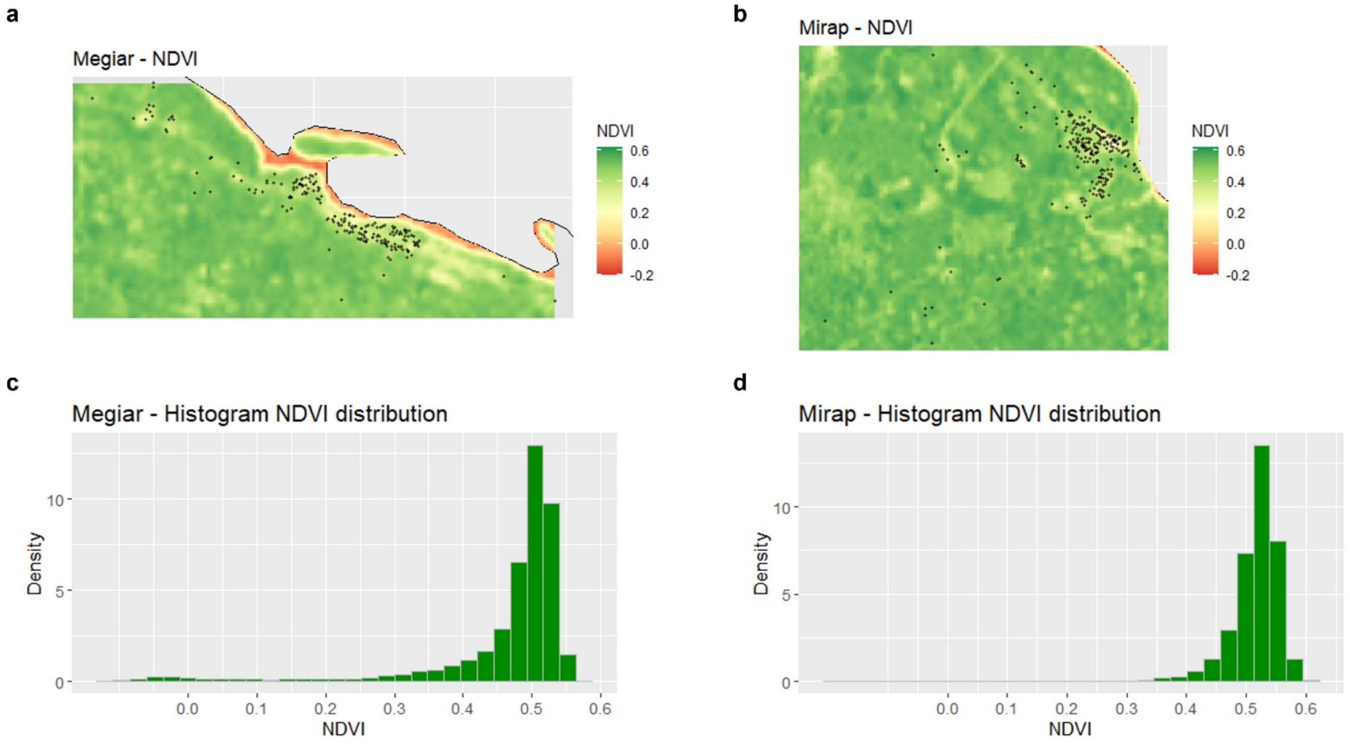

Supplementary Figure 1. Normalised difference vegetation index (NDVI) for (a) Megiar and (b) Mirap. More dense vegetation is denoted by the index being closer to 1 and index closer to 0 signifies little vegetation, while negative values towards -1 denote the presence of water bodies or building structures. Red zones above represent the coastline which has negative NDVI. Black dots represent the sampled households in the study. Histogram distribution of NDVI values for (c) Megiar and (d) Mirap. The vegetation or "greenness" in each village is similar with median NDVI = 0.5 in both Megiar and Mirap. We assumed that NDVI remains fairly constant in 2014 and 2016. Maps were created in R v.3.6.2 (https://www.R-project.org/).

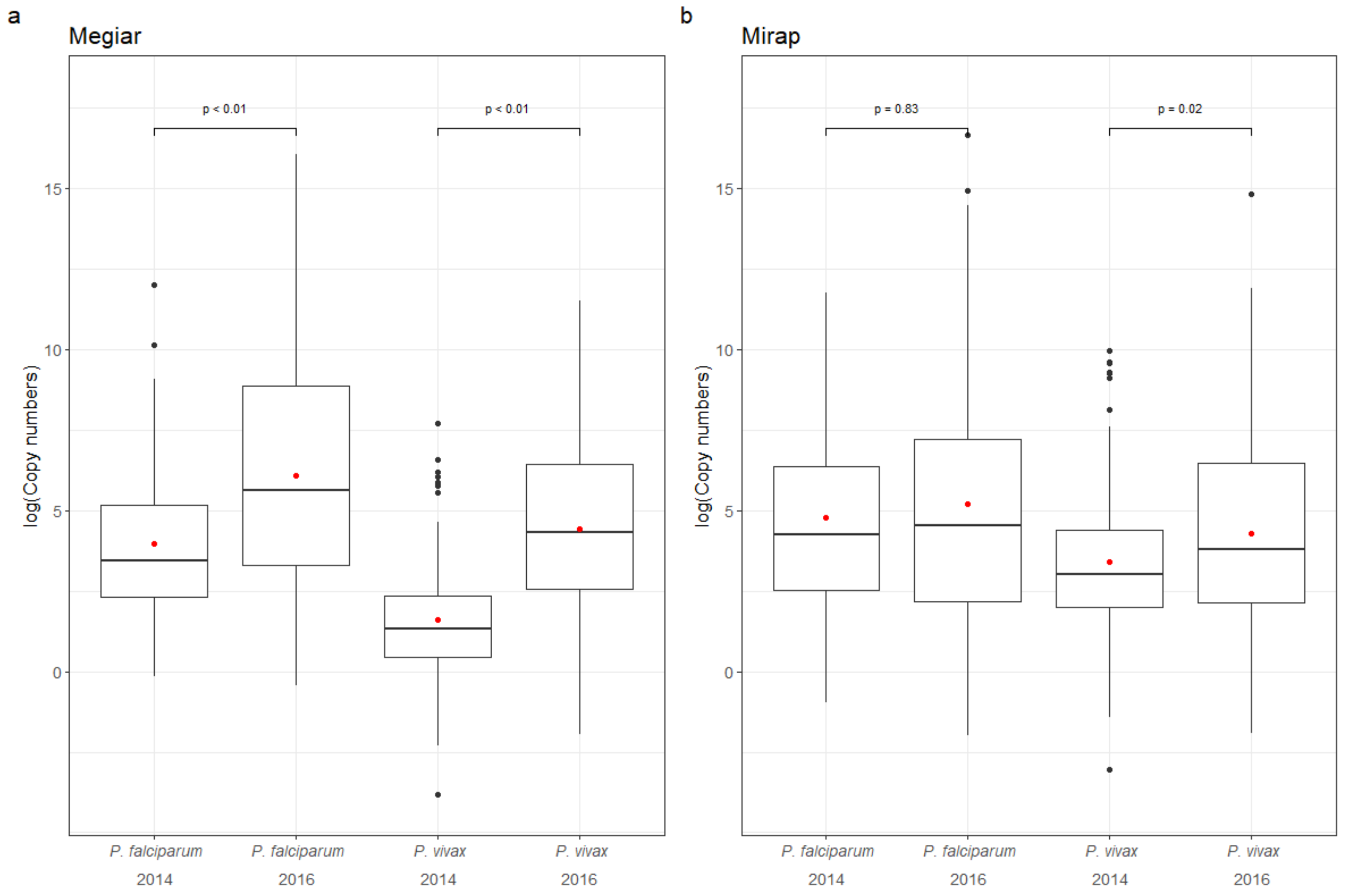

Supplementary Figure 2. Summary of parasite density showing the mean, median, quartile and range by species and year in Megiar (a) and Mirap (b). Mean is shown in red dot and parasite density is expressed as log values of the number of PCR copies as determined from qPCR.

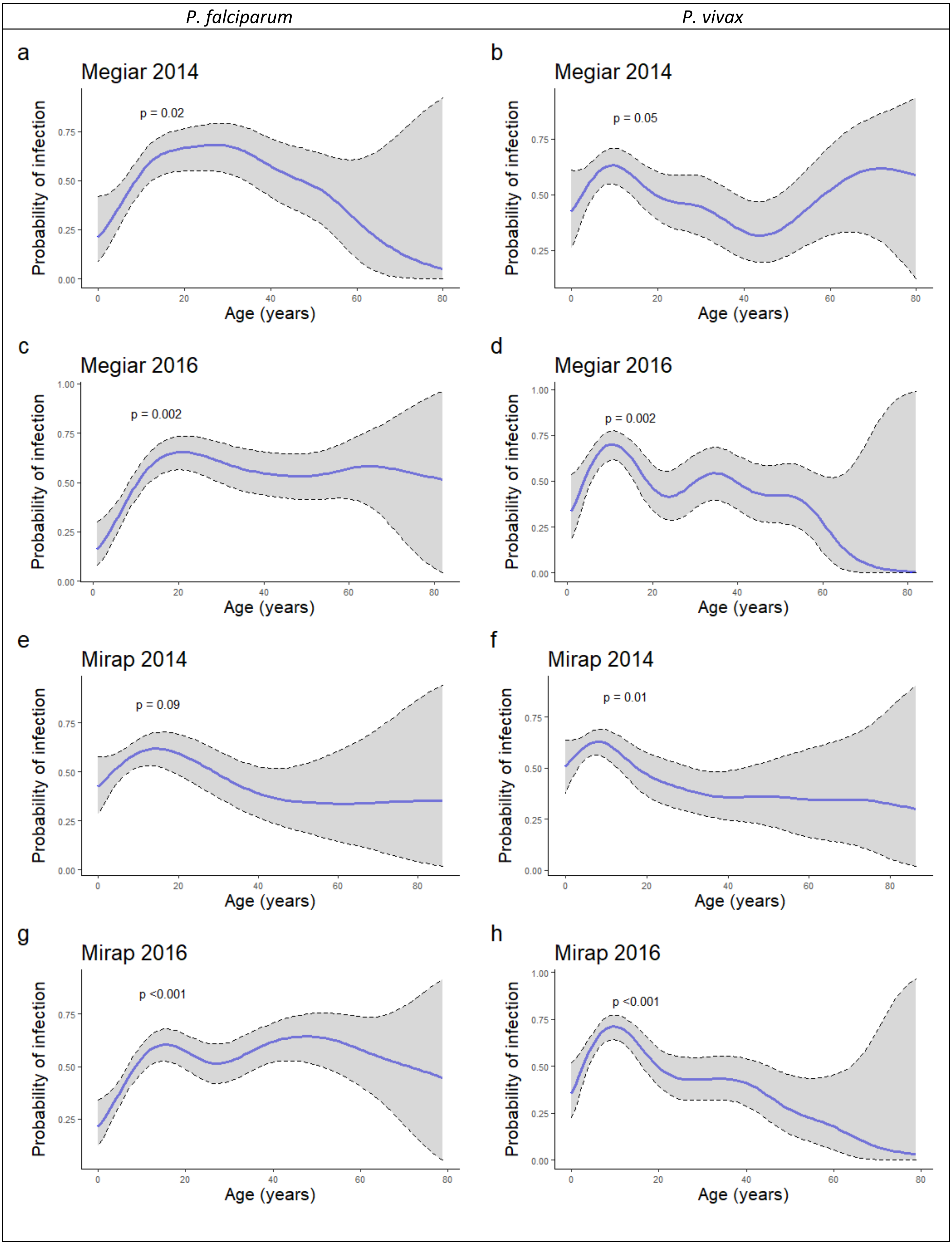

Supplementary Figure 3. Marginal effect of age on probability of infection for *P. falciparum* (left panels: A, C, E, G) and *P. vivax* (right panels: B, D, F, H) in Megiar (A-D) and Mirap (E-H). 95% confidence interval is denoted by the coloured bands. P-values for each of the models are also denoted on the graph. Marginal effect of age is derived from GAM models and the extended GAM models were used in 2016. GAM: generalised additive models.

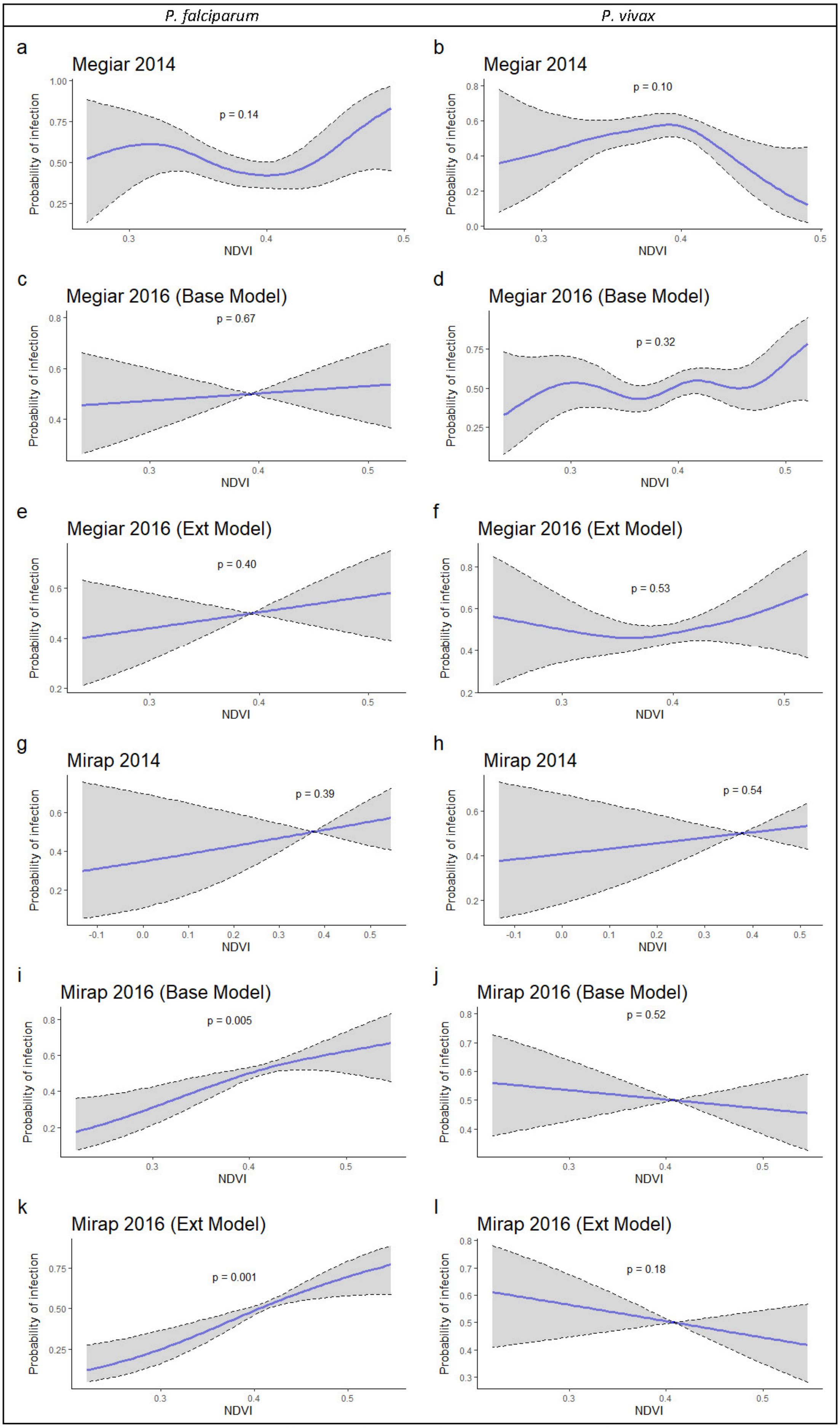

Supplementary Figure 4. Marginal effect of vegetation density on probability of infection for *P. falciparum* (left panels: A, C, E, G, I, K) and *P. vivax* (right panels: B, D, F, H, J, L) in Megiar (A-F) and Mirap (G-L). Base model estimates are shown for 2014. Base and extended model estimates are shown for 2016. 95% confidence interval is denoted by the coloured bands. P-values for each of the models are also denoted on the graph. Marginal effect of vegetation density is derived from GAM models. NDVI: normalised difference vegetation index, GAM: generalised additive model.

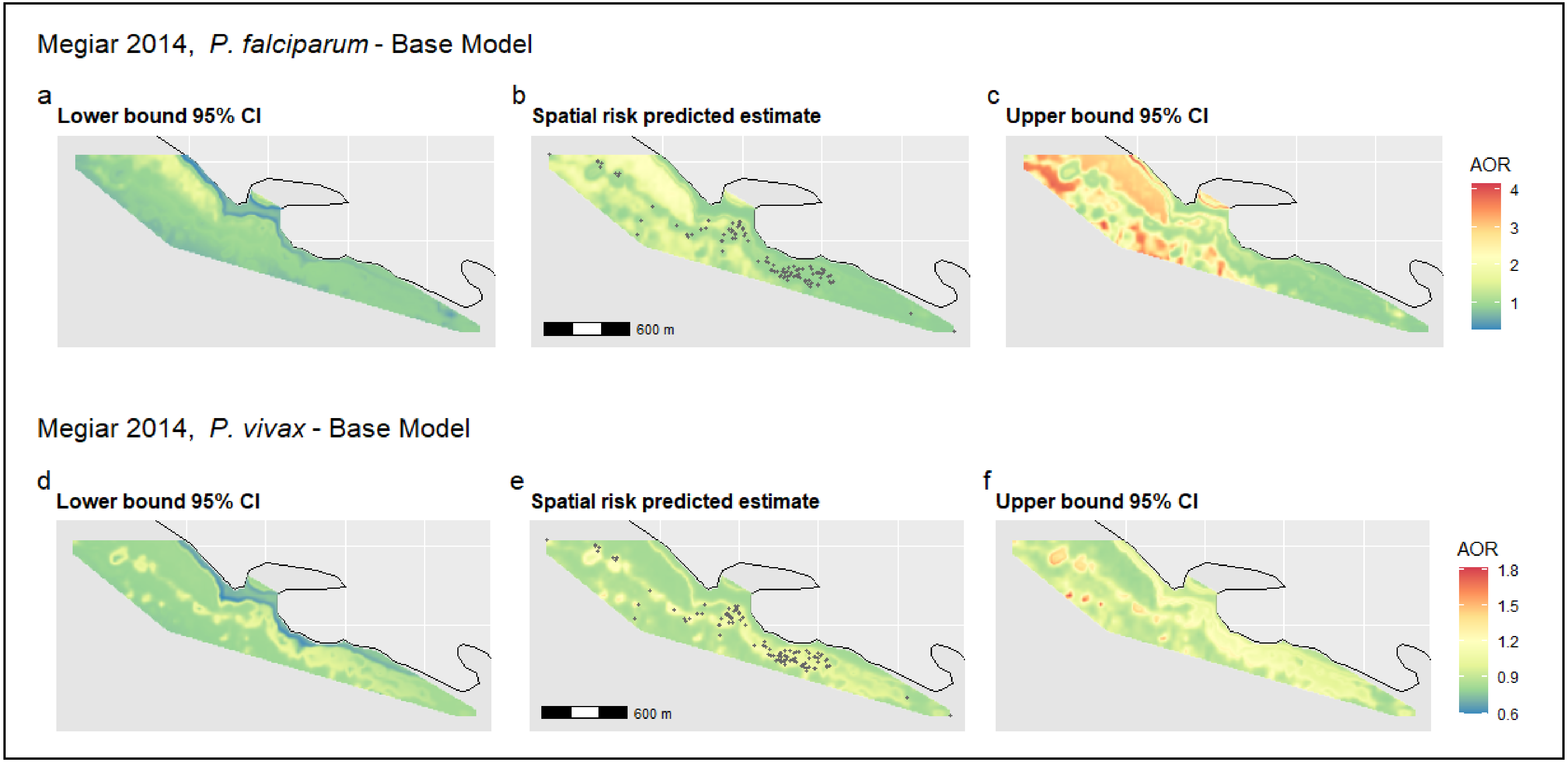

Supplementary Figure 5. Megiar 2014, *P. falciparum* (panels A to C) and *P. vivax* (panels D to F) – Base model spatial effect adjusted odds ratios (AOR) and 95% confidence interval (CI). Point estimates shown in panels B and E. Lower bound CI (panels A and D), Upper bound CI (panels C and F). Spatial risk scale differs from Figure 3 to accommodate the lower and upper bound confidence interval values for this model. Maps were created in R v.3.6.2 (https://www.R-project.org/).

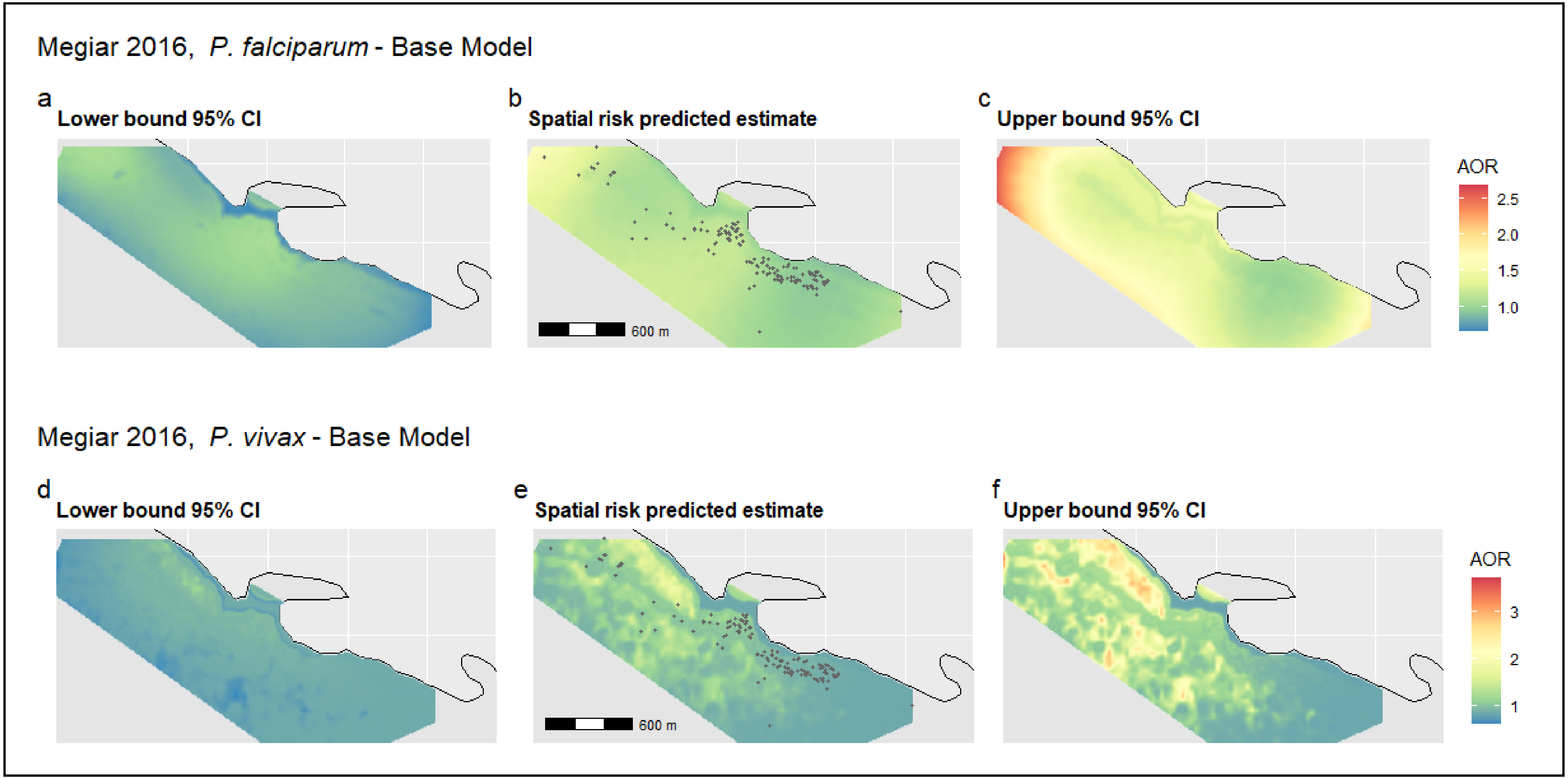

Supplementary Figure 6. Megiar 2016, *P. falciparum* (panels A to C) and *P. vivax* (panels D to F) – Base model spatial effect adjusted odds ratios (AOR) and 95% confidence interval (CI). Point estimates shown in panels B and E. Lower bound CI (panels A and D), Upper bound CI (panels C and F). Spatial risk scale differs from Figure 3 to accommodate the lower and upper bound confidence interval values for this model. Maps were created in R v.3.6.2 (https://www.R-project.org/).

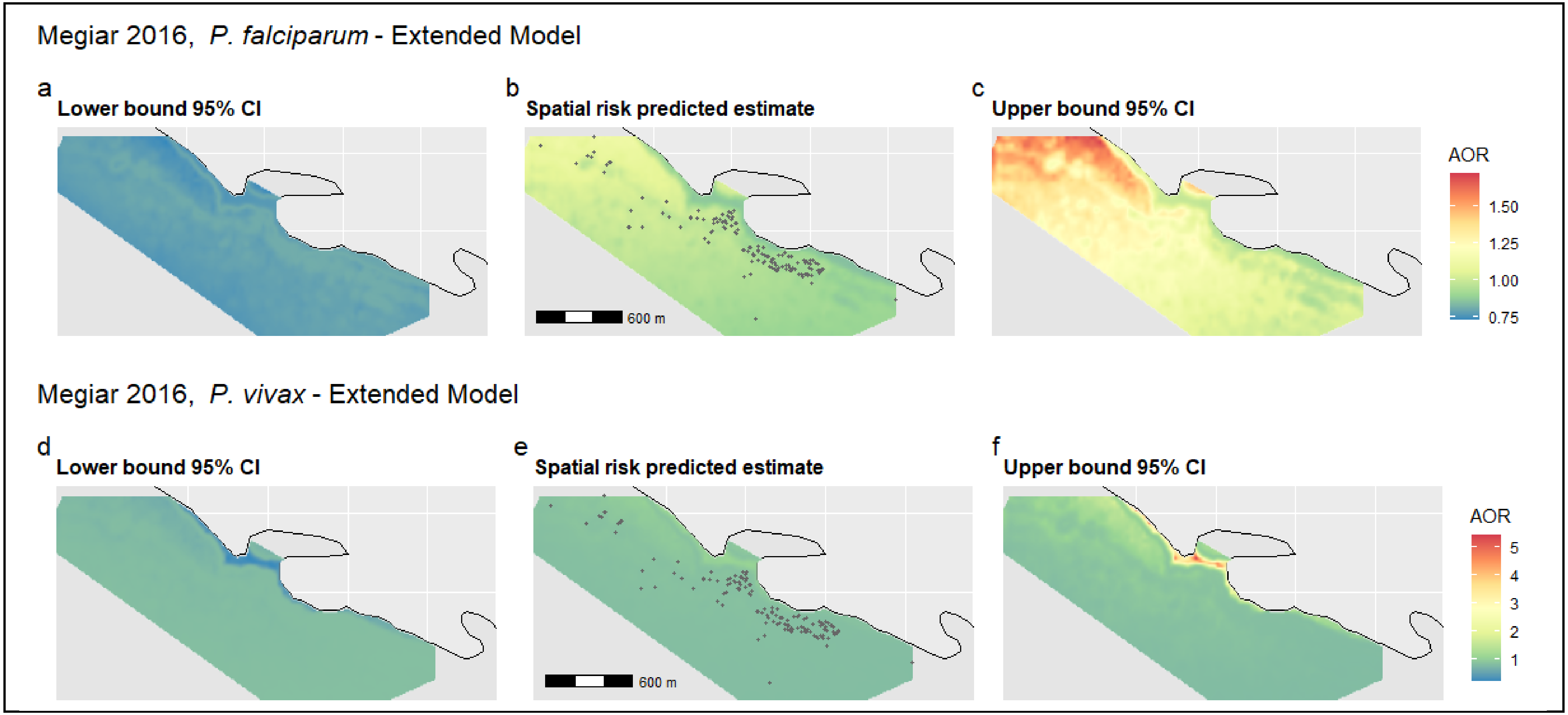

Supplementary Figure 7. Megiar 2016, *P. falciparum* (panels A to C) and *P. vivax* (panels D to F) – Extended model spatial effect adjusted odds ratios (AOR) and 95% confidence interval (CI). Point estimates shown in panels B and E. Lower bound CI (panels A and D), Upper bound CI (panels C and F). Spatial risk scale differs from Figure 3 to accommodate the lower and upper bound confidence interval values for this model. Maps were created in R v.3.6.2 (https://www.R-project.org/).

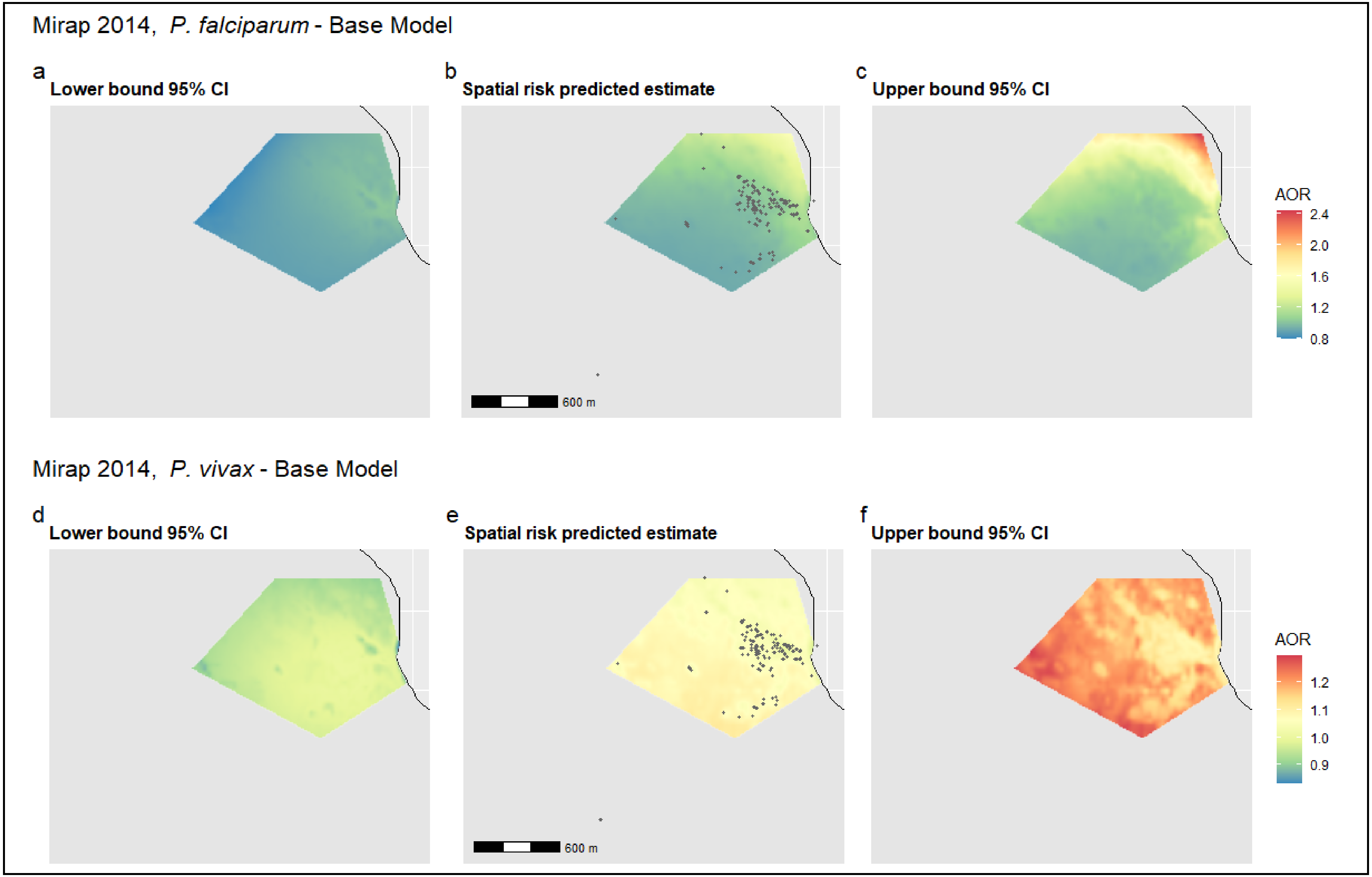

Supplementary Figure 8. Mirap 2014, *P. falciparum* (panels A to C) and *P. vivax* (panels D to F) – Base model spatial effect adjusted odds ratios (AOR) and 95% confidence interval (CI). Point estimates shown in panels B and E. Lower bound CI (panels A and D), Upper bound CI (panels C and F). Spatial risk scale differs from Figure 4 to accommodate the lower and upper bound confidence interval values for this model. Maps were created in R v.3.6.2 (https://www.R-project.org/).

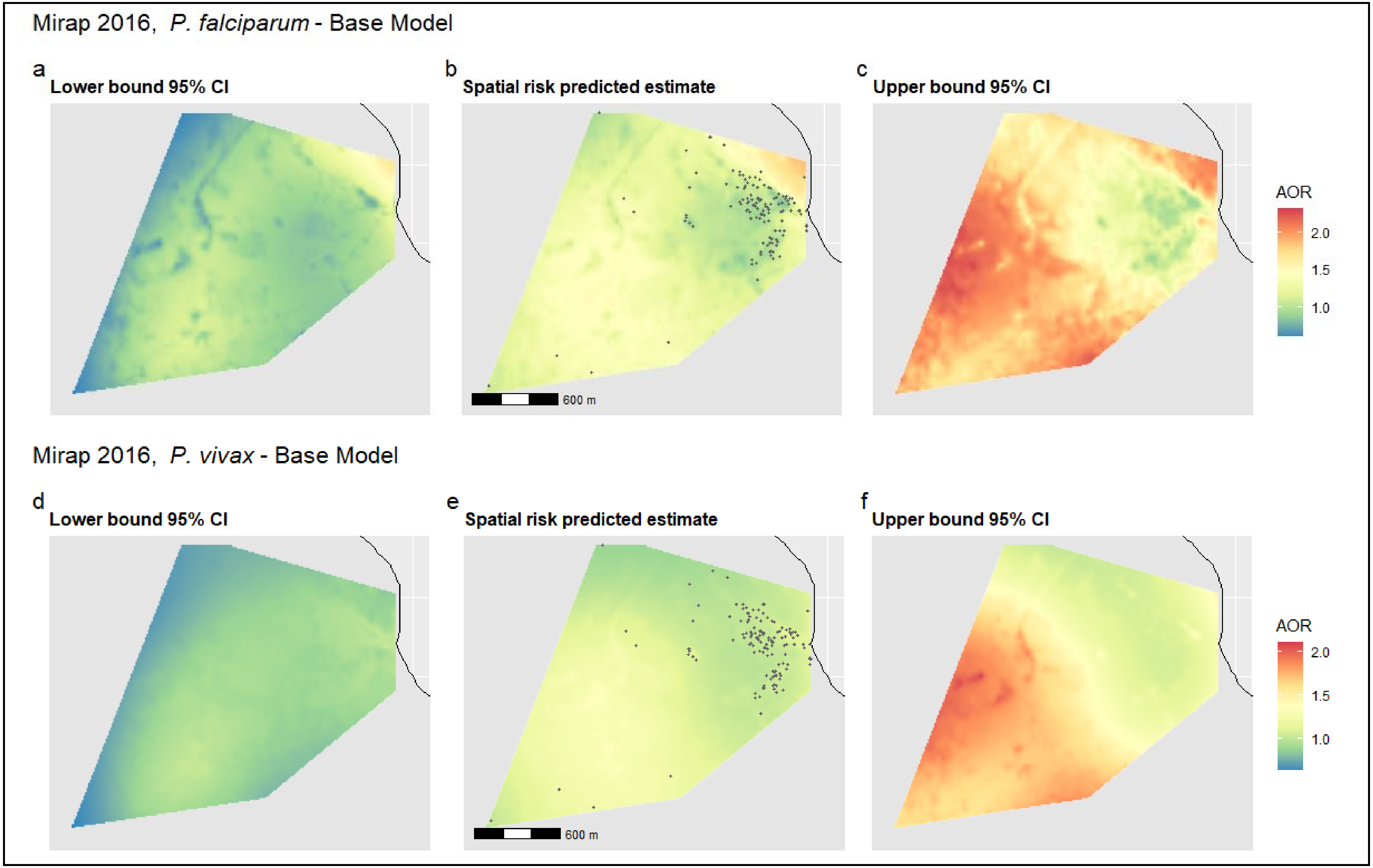

Supplementary Figure 9. Mirap 2014, *P. falciparum* (panels A to C) and *P. vivax* (panels D to F) – Base model spatial effect adjusted odds ratios (AOR) and 95% confidence interval (CI). Point estimates shown in panels B and E. Lower bound CI (panels A and D), Upper bound CI (panels C and F). Spatial risk scale differs from Figure 4 to accommodate the lower and upper bound confidence interval values for this model. Maps were created in R v.3.6.2 (https://www.R-project.org/).

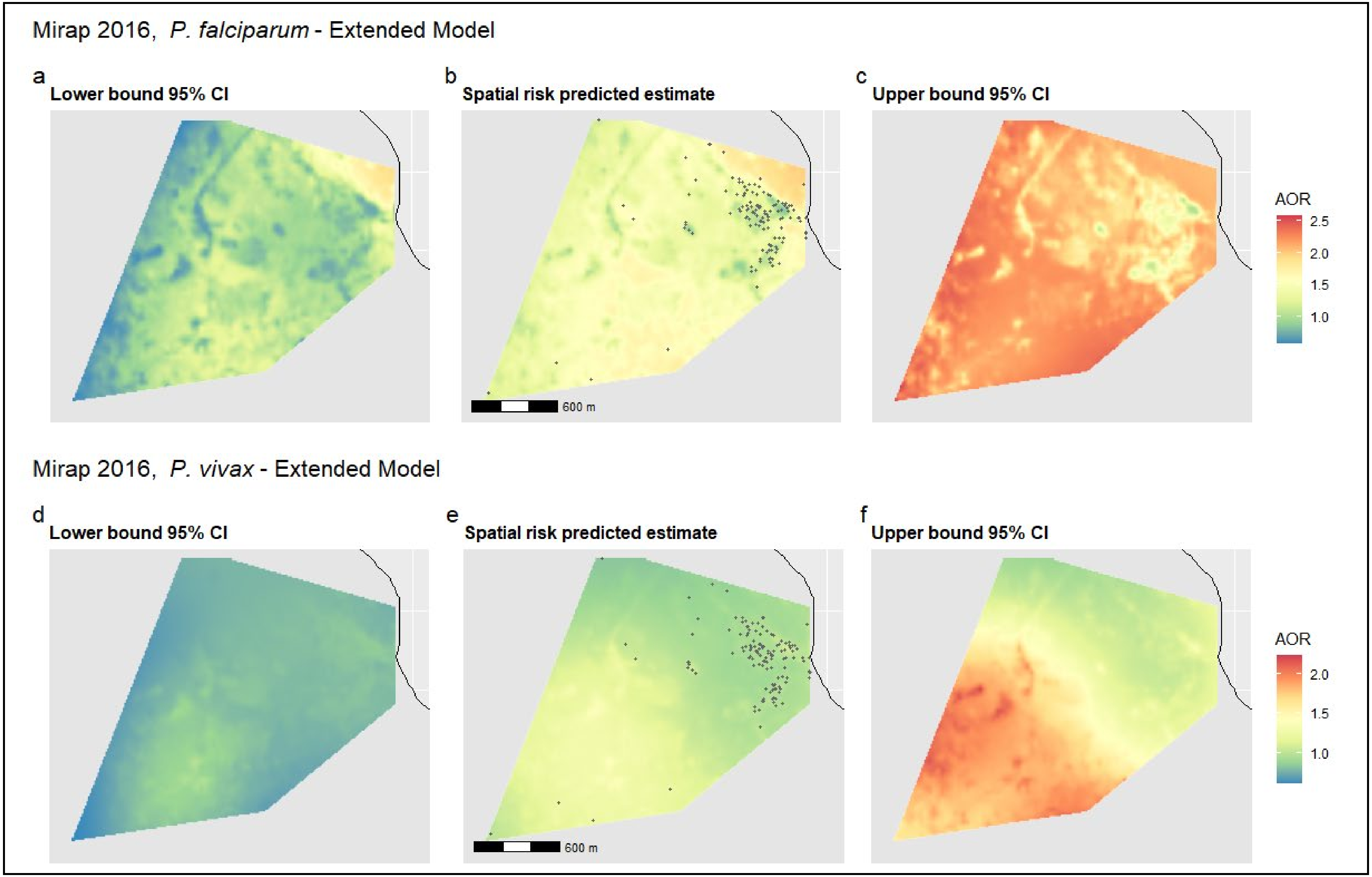

Supplementary Figure 10. Mirap 2014, *P. falciparum* (panels A to C) and *P. vivax* (panels D to F) – Extended model spatial effect adjusted odds ratios (AOR) and 95% confidence interval (CI). Point estimates shown in panels B and E. Lower bound CI (panels A and D), Upper bound CI (panels C and F). Spatial risk scale differs from Figure 4 to accommodate the lower and upper bound confidence interval values for this model. Maps were created in R v.3.6.2 (https://www.R-project.org/).

Characteristics	Megiar			Mirap		
	**2014 (%)**	**2016 (%)**	**p-value[a]**	**2014 (%)**	**2016 (%)**	**p-value**
**Sample size**	509	601		529	672	
**Number of households**	121	132		120	127	
**Sex - Male**	233 (45.8)	278 (46.3)	0.873	236 (44.6)	328 (48.8)	0.148
**Median age (years) [inter-quartile range]**	16.8 [7, 35]	19.0 [9, 39]	0.169	13.3 [5, 32]	16.0 [6, 33]	0.137
**Age groups[b]:**			0.052			0.063
**0-5yrs**	112 (22.0)	90 (15.0)		152 (28.7)	150 (22.3)	
**6-10yrs**	69 (13.6)	95 (15.8)		80 (15.1)	111 (16.5)	
**11-15yrs**	63 (12.4)	73 (12.2)		61 (11.5)	68 (10.1)	
**16-20yrs**	51 (10.0)	54 (9.0)		34 (6.4)	58 (8.6)	
**21-25yrs**	36 (7.1)	50 (8.3)		27 (5.1)	50 (7.4)	
**≥26yrs**	178 (35.0)	239 (39.8)		171 (32.6)	235 (35.0)	
**Bednet usage[b]**	417/505 (82.6)	477/588 (81.1)	0.535	451/527 (85.6)	629/670 (93.9)	<0.001

Supplementary Table 1. Summary of the demographic characteristics of surveyed population in Megiar and Mirap in 2014 and 2016. Data presented as number and percentage in round parenthesis. Interquartile range if applicable is presented in square parenthesis.

a p-value based on chi-square test comparing counts of variable between 2014 and 2016 within each village.

b Due to missing values, denominators are smaller than sample size.

Characteristics	Megiar	Mirap	p-value[a]
**Total population (2016 Census)**	991	1283	
**Total number of households (2016 Census)**	171	217	
**Number of households surveyed**	132	127[b]	
**Household head highest school level**			0.072
Grade 6 and below	52 (39.4)	66 (52.4)	
Grade 8	35 (26.5)	28 (22.2)	
Grade 10	29 (22.0)	26 (20.6)	
Grade 12 and above	16 (12.1)	6 (4.8)	
**Household main income source**			<0.001
Wage job (private/govt/public sector)	19 (14.4)	2 (1.6)	
Self-employed / Family business / Marketing / Street-seller	23 (17.4)	15 (11.9)	
Gardening / Farming / Fishing / Timber	90 (68.2)	109 (86.5)	
**Main material of floor**			0.003
Earth / Sand / Palm / Bamboo / Grass	81 (61.4)	53 (42.1)	
Wood	48 (36.4)	72 (57.1)	
Cement / Tiles	3 (2.3)	1 (0.8)	
**Main material of wall**			<0.001
Bamboo / Pitpit	62 (47.0)	22 (17.5)	
Sago	37 (28.0)	97 (77.0)	
Wood / Plywood / Masonite	29 (22.0)	1 (0.8)	
Cement / Bricks / Iron sheets	4 (3.0)	6 (4.8)	
**Main material of roof**			<0.001
Thatched grass / Sago palm leaves	61 (46.2)	113 (89.7)	
Corrugated iron	71 (53.8)	13 (10.3)	
**Windows type**			<0.001
No windows / Not screened	26 (19.7)	64 (50.8)	
Partially screened	51 (38.6)	46 (36.5)	
All screened	55 (41.7)	16 (12.7)	
**Main source of drinking water**			<0.001
Surface water (river, pond, stream, etc.)	38 (28.8)	10 (7.9)	
Well (open / protected; public / private)	0	100 (79.4)	
Own water tank / Piped into dwelling	46 (34.9)	12 (9.5)	
Public tap / Piped into neighbourhood	48 (36.4)	4 (3.2)	

Characteristics	Megiar	Mirap	p-value[a]
**Household toilet facility**			<0.001
**No facility / bush / seashore**	31 (23.5)	110 (87.3)	
**Shared pit latrine / flushed toilet**	43 (32.6)	3 (2.4)	
**Own pit latrine / flushed toilet**	58 (43.9)	13 (10.3)	
**Median SES index [inter-quartile range]**	-0.392 [-1.367, 0.703]	-0.471 [-0.801, -0.029]	0.16

Supplementary Table 2. Summary of the household characteristics of surveyed households in Megiar and Mirap in 2016. Data presented as number and percentages in round parenthesis. Interquartile range if applicable is presented in square parenthesis.

a p-value is based on chi-square test comparing counts of household characteristics between Megiar and Mirap in 2016.

b Due to missing values for 1 household in this village, all percentages are based on a denominator of 126 households.
